# Analysis of volatile compounds emitted from white champaca flowers (*Michelia alba* D.C.) via HS-GC-MS and SPME-GC-MS technology

**DOI:** 10.3389/fpls.2026.1780030

**Published:** 2026-04-01

**Authors:** Xiangyang Guo

**Affiliations:** 1 College of Tea and Food Science, Xinyang Normal University, Xinyang, China; 2 College of Chemistry and Environmental Engineering, Shenzhen University, Shenzhen, China

**Keywords:** aroma profiles, headspace (HS) extraction, relative odor activity value (ROAV), solid-phase microextraction (SPME) extraction, volatile compounds, white champaca flowers (*Michelia alba* D.C.)

## Abstract

The utilization of white champaca (*Michelia alba* D. C.) is largely dictated by the physical state of its flowers (fresh or dried), which significantly impacts aroma characteristics and application scope. However, a comprehensive comparison of the aroma profiles between fresh and dried white champaca flowers remains scarce. This study aims to bridge this gap by comprehensively analyzing the volatile profiles of fresh and dried flowers using solid-phase microextraction (SPME) and headspace (HS) extraction coupled with gas chromatography–mass spectrometry (GC-MS) analysis, principal component analysis (PCA), and relative odor activity value (rOAV) determination. A total of 207 volatiles were identified, with the HS method extracting the highest number of compounds (150) from dried flowers, followed by SPME of dried (83) and fresh (78) flowers. Alkene and alcohol compounds dominated dried flowers, whereas esters and heterocyclic compounds were prevalent in fresh flowers. Significant differences on aroma characteristics were observed among the three flower samples, which were well distinguished by PCA. Furthermore, 15 volatiles (rOAV > 1) had a positive contribution for the overall aroma of flower samples, of which methyl anthranilate with an rOAV value of 100 was the primary contributor to the fruity note in fresh and SPME-dried samples, while linalool (rOAV = 100) imparted the dominant floral scent in the HS-dried samples. These findings provide a theoretical basis for the targeted utilization of white champaca in the fragrance and flavor industries.

## 1 Introduction

With the improvement of living standards, consumers have become increasingly concerned about natural products. Because of its elegant floral fragrance and beautiful appearance, white champaca (*Michelia alba* D.C.) has long been popular and appreciated in China and Southeast Asia.

As an annual flowering plant widely cultivated in tropical and subtropical regions, white champaca is a common landscape tree. It effectively improves thermal comfort and microclimatic performance in subtropical regions (Zheng et al., 2018), mitigates the urban heat island effect (Zheng et al., 2020a), provides cooling in hot and humid climates (Zheng et al., 2021), and significantly impacts urban ventilation (Zheng et al., 2020b). Beyond its ecological benefits, white champaca possesses edible value (Zheng et al., 2018) and preservation properties (Chen et al., 2021), along with excellent pharmacological activities, including anti-acne (Asnaashari et al., 2023), antioxidant (Zheng et al., 2018), antifungal (Suhem et al., 2017; Matan et al., 2023), antibacterial (Songsamoe et al., 2021), antidiabetic (Cheng et al., 2022), and larvicidal effects (Sheng et al., 2020). Historically, it has been used to treat syphilis, malaria, fever, and gonorrhea, and even used as an abortive agent (Lee et al., 2014). Moreover, white champaca has garnered increasing attention for its outstanding aroma, characterized by a high-intensity floral scent. It is widely used to scent green teas (Hou et al., 2024) and is a favored ingredient in the fragrance industry for blending perfumes, cosmetics, and daily chemical products (Wang et al., 2024).

Aroma is a critical factor influencing flower quality, sensory characteristics, and economic value, ultimately determining consumer preference (Wang et al., 2021). Volatile compounds constitute the material basis of flavor and aroma in aromatic plants, and the pharmacological properties of white champaca may be related to the composition and proportion of volatile components (Suhem et al., 2017; Matan et al., 2023). Previous research reported that linalool is the most abundant volatile compound contributing to floral odor in white champaca (Xia et al., 2010; Cheng et al., 2022) and may be a key factor in enhancing the antifungal activity of its essential oil (Suhem et al., 2017). In addition, caryophyllene imparting woody odor is also the main compound in white champaca essential oil, which has the effect of inhibiting the growth of fungus (Suhem et al., 2017). Therefore, analyzing the volatile compositions of white champaca flowers is of great significance to reveal its aroma characteristics and explore its functional activities. Generally, fresh flowers are directly harvested for scenting tea or extracting essential oils. However, their high moisture content limits long-distance transport and long-term preservation. Dried flowers alleviate these logistical issues but are often considered inferior in aroma quality without scientific validation. Regarding the analysis of volatile compounds, existing studies predominantly focus on essential oils, extracts, or concretes derived from flowers or leaves (Ueyama and Hashimoto, 1992; Kamli et al., 2024; Wang et al., 2024), whereas investigations into the headspace (HS) aroma profiles of fresh versus dried flowers are scarce. Furthermore, there is a lack of comparative research analyzing how the drying process alters the volatile fingerprint and key odorants of white champaca.

Aroma compounds are typically obtained using solid-phase microextraction (SPME) or HS extraction. While SPME is a convenient, artifact-free method widely applied for enriching volatiles from plant matrices (Issa et al., 2020), HS is particularly efficient for detecting low and middle boiling point components (Guo et al., 2019), which is commonly used to analyze the volatile compounds in natural plants and flowers (Virgiliou et al., 2021). Despite the common use of these techniques, existing literature predominantly focuses on the analysis of essential oils or extracts, leaving a significant gap in understanding how the physical state of the flower (fresh *vs*. dried) and the choice of extraction methodology interact to shape the aroma profile. To address this, the present study provides a systematic comparison of volatile profiles in fresh and dried white champaca flowers using both SPME and HS extraction coupled with gas chromatography–mass spectrometry (GC-MS) analysis, principal component analysis (PCA), and relative odor activity value (rOAV) determination. The novel contribution of this work lies in the comprehensive quantification of volatile transformations induced by drying and the precise identification of state-specific key odorants. Specifically, this research investigates how the drying process alters the chemical composition and relative abundance of volatile compounds, identifies the key odorants responsible for the characteristic aroma of dried flowers compared to fresh ones, and evaluates the efficacy of different extraction methods in capturing the authentic aroma profile of dried floral products. By elucidating these aspects, this study aims to provide a precise theoretical basis for the industrial processing and application of white champaca flowers.

## 2 Materials and methods

### 2.1 Reagents and materials

The fresh flowers of white champaca (*M. alba* D.C.) were harvested in May 2019 from Guangde City (Anhui province, China). Some of the fresh flower samples were directly subjected to volatile detection, and the remaining fresh flowers were freeze-dried using a freeze dryer (ALPHA 1–4 LD plus, Martin Christ Freeze Dryer, Germany). Detailed protocols for sample collection and freeze-drying are provided in the **Supplementary Methods**.

### 2.2 Aroma extraction by solid-phase microextraction

The SPME method was applied for the extraction of volatile compounds from fresh (FF-SPME) and dried (DF-SPME) flowers of white champaca, which referred to the method recorded by a previous study (Guo et al., 2019) with some modifications. Detailed protocols for parameter optimization are provided in the **Supplementary Methods**. In detail, the white champaca flower samples (5.0 g of fresh flowers or 2.0 g of dried flowers) were weighted and transferred into a 100-mL beaker flask, which was sealed with foil. Then, the prepared flower samples were placed into a water bath (45°C) for equilibration for 6 min. An SPME fiber coated with a 50/30-μm layer of DVB/CAR/PDMS (Supelco, Inc., Bellefonte, PA, USA) was used to extract the volatile compounds at 45°C for 45 min. Thereafter, the SPME fiber was withdrawn and loaded to a GC injector with a split ratio of 10:1 for the desorption (5 min) of the volatile compounds.

### 2.3 Aroma extraction by headspace

The volatile compounds of dried flowers in white champaca were also extracted using the HS method (DF-HS) described by Guo et al. (2025b) with a slight modification. Dried flower samples (2.0 g) were accurately weighted and placed into a 20-mL HS vial, and the vial was subsequently covered using an HS auto-loading crimp cap with septa. A GC (Agilent 7890A, Santa Clara, CA, USA) connected to an Agilent 7697A HS injector (Santa Clara, CA, USA) was used to analyze the volatile compounds in dried flowers of white champaca. The oven temperature, transfer-line temperature, and manifold temperature were set at 120°C, 150°C, and 140°C, respectively. The injection time and the incubation time were 60 s and 20 min, respectively.

### 2.4 GC-MS analysis of volatile composition

In the present study, an Agilent 7890A-5975C model GC-MS (Agilent, Santa Clara, CA, USA) was used to analyze the volatile compounds, coupled to a DB-5 fused silica capillary column (60 m × 0.25 mm × 0.25 μm, J&W, Folsom, CA, USA) for the separation of volatile components, which was proposed by Guo et al (Guo et al., 2019). The column oven temperature was set at 50°C and maintained for 5 min, raised at a rate of 3°C/min to 210°C and held for 3 min, then increased to 230°C at 10°C/min and maintained for 2 min, subsequently raised at a rate of 10°C/min to 280°C, and held for another 10 min. The carrier gases were helium gas (37 cm/s) with a purity ≥ 99.999%. The mass spectrometer was operated at EI ionization style (positive ion, 70 eV). The transfer-line and ion source temperatures were 150°C and 250°C, respectively. The mass spectra were acquired in full scan mode with a range of 30–500 amu. Retention indices (RIs) were determined from the retention time of C5–C28 *n*-alkanes by linear interpolation. The peak was deconvoluted, and the identification of the chemical compounds was based on comparison with the NIST Mass Spectral Library (2017 version), RI, references, and in-house database. Some volatile compounds were positively identified with those of standard compounds. The amounts of the identified volatile compounds were determined as the percentage of total peak areas and were expressed as relative amount (%).

### 2.5 Determination of relative odor activity value

To further understand the contribution of volatile compounds to the overall aroma of white champaca flowers, rOAV was determined in this study (Guo, 2025). The calculation of rOAV is based on **Equation 1**, as shown below.

(1) $$\text{rOA}(\%)=\frac{OAVi}{OAVmax}\times 100=\frac{\frac{C_{i}}{T_{i}}}{\frac{Cmax}{Tmax}}\times 100=\frac{\frac{Ra_{i}}{T_{i}}}{\frac{Ramax}{Tmax}}\times 100$$

where OAVi is the odor activity value (OAV) of the volatile component, which is determined by using the ratio of the concentration (*C_i_*) of each volatile component to its corresponding odor threshold value (*T_i_*) in air. “max” is the volatile component that has the highest OAV value. Ra*_i_* is the relative amount (%) of the volatile component in white champaca flower samples (fresh and dried flowers). The rOAV of the volatile component, which has the OAV_max_, is regarded as 100.

### 2.6 Statistical analysis

Statistical analysis was processed by SPSS Statistics (SPSS 22.0 for Windows, SPSS Inc., Chicago, IL). One-way analysis of variance (ANOVA) and Duncan test with *p*-value <0.05 was considered to be statistically significant. All experiments were conducted in triplicate. Method validation focused on precision and detection capability, given the semi-quantitative nature of the peak area normalization method. Repeatability was confirmed with RSD values for major peaks below 10% across three replicates. The limit of detection (LOD) was established at a signal-to-noise (S/N) ratio of 3:1.

## 3 Results and discussion

### 3.1 Volatile profiles of white champaca from fresh and dried flowers

**Figure 1:**
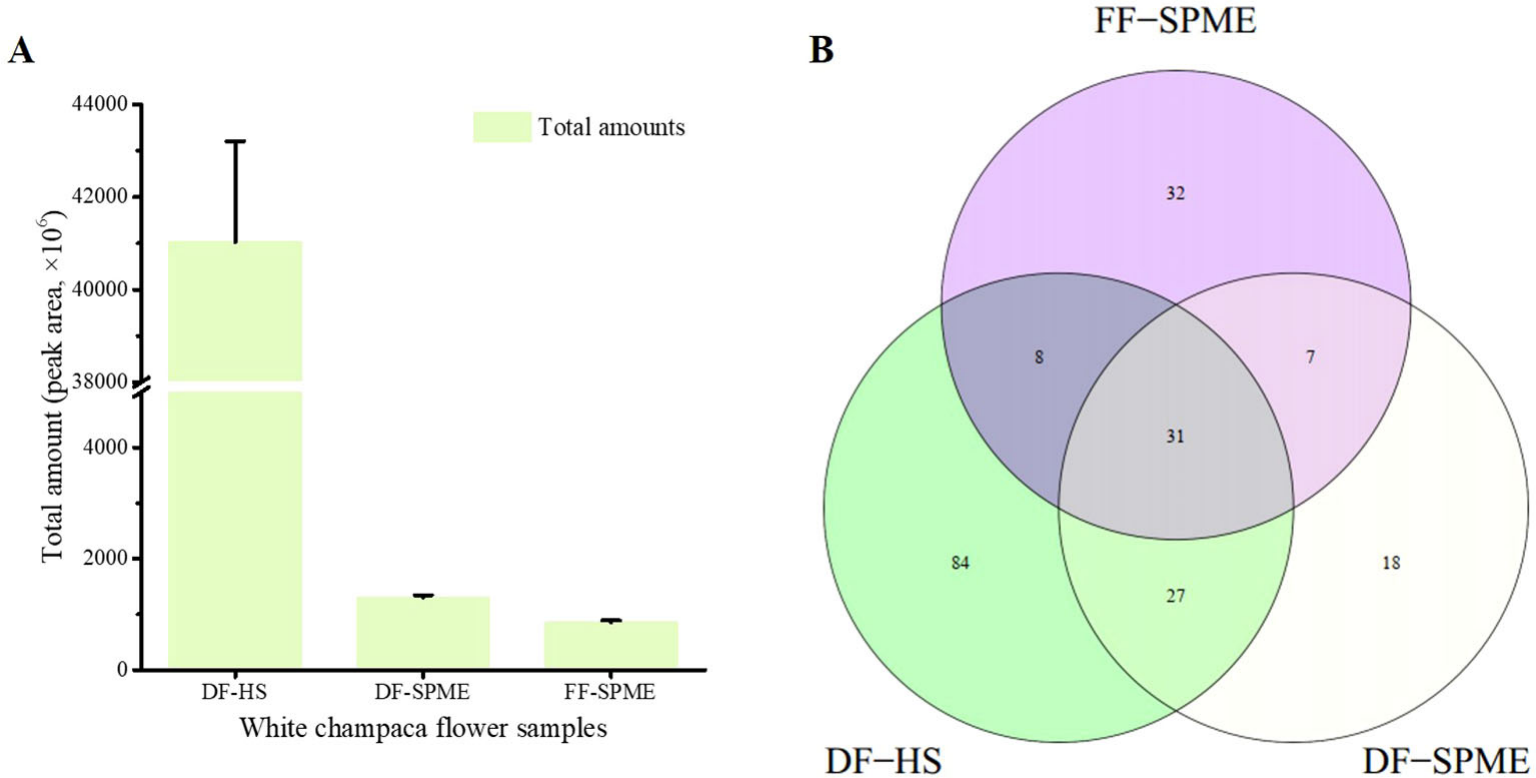
Volatile profiles of white champaca from fresh and dried flowers. **(A)** The total amounts of volatiles in white champaca from fresh flowers (FF-SPME) and dried flowers (DF-SPME, DF-HS). **(B)** The Venn diagram of different volatile compounds in white champaca from fresh and dried flowers.

A total of 207 volatile components were identified across fresh and dried white champaca flowers (**Supplementary Table 1**), in which 31 volatiles were commonly found among three samples (**Figure 1A**). Among these, 128 volatiles had relative amounts exceeding 0.10% (**Table 1**). Specifically, 32, 18, and 84 volatiles were solely identified in FF-SPME, DF-SPME, and DF-HS samples, respectively. Dried flowers exhibited a higher number and total amount of volatiles compared to fresh flowers, with DF-HS showing significantly higher total amounts than both FF-SPME and DF-SPME (**Figure 1B**).

**Table 1:** The identified volatiles with relative amounts higher than 0.1% in white champaca from fresh and dried flowers.

No.	Volatile compounds	RT (min)	RI	ID ^ζ^	Odor quality ^ψ^	Relative amount (%)			Threshold (mg/m^3^) ^#^
						DF-HS	FF-SPME	DF-SPME	
1	Butanal	2.05	576	MS,RI	Green, musty, pungent	0.12±0.02	nd	nd	0.1
2	Acetic acid	2.23	595	MS,RI	Sour	0.40±0.04	nd	nd	0.013
3	3-Methylbutanal	2.79	653	MS,RI	Malty, fruity, apple/peach-like	0.34±0.01	nd	nd	0.00035
4	2-Methylbutanal	2.96	670	MS,RI	Pungent, coffee/cocoa-like, fruity	0.98±0.13	nd	nd	0.1
5	Ethyl 2-methylpropanoate	4.51	756	MS,RI	Fruity, creamy	2.12±0.30	nd	nd	0.00011
6	Methyl 2-methylbutanoate	4.93	775	MS,RI	Fruity (pungent), fatty, creamy, green	0.34±0.04	nd	nd	8.76
7	Hexanal	5.60	803	MS,RI,S	Grassy, green, fresh, fatty	0.30±0.06	nd	0.15±0.02	0.23
8	Methyl 2,3-dimethylbutanoate	6.29	822	MS,RI	——	nd	1.37±0.14	nd	n.f.
9	Ethyl 2-methylbutanoate	7.23	848	MS,RI	Fruity, peely (apple, pineapple)	2.66±0.14	nd	0.14±0.0	11.7
10	2-Hexenal	7.27	849	MS,RI,S	Grassy, herbal	nd	nd	0.25±0.04	0.48
11	2-Methylbutanoic acid	7.78	863	MS,RI	Pungent, goat cheese-like, fruity	1.04±0.07	nd	2.60±0.44	0.02
12	2-Heptanone	8.74	890	MS,RI	Pear-like, fruity	0.63±0.05	nd	nd	0.0035
13	(*E,E*)-2,4-Hexadienal	9.57	910	MS,RI,S	Green, sweet, fruity	nd	nd	0.20±0.02	0.0018
14	2-Methylhexanoic acid	9.92	918	MS,RI	Soury, fatty, roasted food-like	0.29±0.03	nd	nd	2700
15	Butyrolactone	10.38	928	MS,RI	Acetone-like	0.28±0.03	nd	nd	>1000
16	*α*-Pinene	10.50	931	MS,RI,S	Pine-like	0.81±0.02	nd	nd	0.1
17	Camphene	11.23	947	MS,RI	Camphor-like	0.24±0.01	nd	nd	30
18	Benzaldehyde	11.83	960	MS,RI,S	Almond-like, fruity, cherry-like, powdery	0.10±0.02	0.24±0.03	0.49±0.02	0.085
19	*β*-Thujene	12.33	971	MS,RI	Woody, spicy, citrus	0.40±0.03	nd	nd	n.f.
20	*β*-Pinene	12.53	975	MS,RI,S	Pine-like	0.94±0.23	nd	nd	0.18
21	*β*-Myrcene	13.18	989	MS,RI,S	Woody, resinous, musty, balsamic, ethereal	0.27±0.05	nd	0.12±0.01	0.1125
22	Ethyl hexanoate	13.69	1000	MS,RI,S	Green apple	0.33±0.01	nd	0.06±0.01	70
23	(*E,E*)-2,4-Heptadienal	14.21	1011	MS,RI,S	Fatty, green, oily, cinnamon-like	nd	nd	0.15±0.0	0.057
24	3-Pyridinecarbonitrile	14.26	1012	MS,RI	Burnt, cocoa-like	0.01±0.0	nd	0.56±0.09	n.f.
25	*α*-Terpinene	14.42	1015	MS,RI,S	Citrusy, woody, lemon-like	0.10±0.02	nd	nd	7.9
26	*o*-Cymene	14.81	1023	MS,RI	Aromatic	0.17±0.01	nd	nd	0.005
27	Limonene	15.02	1028	MS,RI,S	Citrus, lemon, orange,green, etherel	0.26±0.03	nd	0.06±0.01	0.21
28	Eucalyptol	15.20	1031	MS,RI	Camphor-like, herb	1.76±0.26	nd	0.16±0.01	0.15
29	*trans*-*β*-Ocimene	15.51	1038	MS,RI,S	Warm, floral, herbal, sweet	3.16±0.24	nd	0.31±0.31	0.0187
30	Benzeneacetaldehyde	15.79	1043	MS,RI,S	Floral, rose, cherry-like	0.11±0.01	nd	0.07±0.0	6.3
31	*α*-Ocimene	16.04	1049	MS,RI,S	Green, woody, tropical	4.33±0.39	0.37±0.04	1.02±0.01	0.0187
32	*γ*-Terpinene	16.46	1057	MS,RI,S	Citrus, lemon-like, woody, spicy, juicy	0.65±0.19	nd	nd	55.0
33	Linalool oxide 2 (trans, furanoid)	17.31	1074	MS,RI,S	Sweet, floral, creamy	0.27±0.04	nd	nd	60
34	*α*-Terpinolen	17.75	1084	MS,RI	Fresh, woody, citrus, pine-like, sweet	0.11±0.0	nd	nd	200
35	Linalool oxide 1 (cis, furanoid)	17.88	1086	MS,RI,S	Sweet, floral, creamy	0.28±0.07	nd	0.07±0.0	100
36	Methyl benzoate	18.37	1096	MS,RI	Floral, fruity	nd	9.38±0.36	0.12±0.0	0.0015
37	Linalool	18.69	1103	MS,RI,S	Floral, sweet	52.10±2.14	1.39±0.30	26.29±0.26	0.0024
38	Phenylethyl alcohol	19.79	1126	MS,RI,S	Floral, rose-like	0.67±0.05	3.08±0.10	1.02±0.04	0.021
39	(4*E*,6*Z*)-*allo*-Ocimene	20.07	1131	MS,RI	Sweet, floral, nutty, herbal, peppery	0.04±0.01	0.08±0.02	0.18±0.01	0.01
40	Cosmene	20.13	1133	MS,RI	Herb, citrus	0.03±0.0	0.26±0.04	0.33±0.05	n.f.
41	Benzyl nitrile / Phenylacetonitrile	20.61	1143	MS,RI	Almond-like	0.03±0.0	0.38±0.08	0.07±0.01	1200
42	Ethyl benzoate	21.98	1171	MS,RI	Holly-like, green , sweet	nd	0.69±0.05	0.35±0.06	0.0006
43	Linalool oxide (pyranoid)	22.08	1173	MS,RI,S	Floral, honey-like	0.26±0.01	nd	0.11±0.01	3600
44	Isoborneol	22.16	1175	MS,RI	Camphor-like	0.17±0.01	nd	nd	16
45	Linalool oxide (pyranoid)	22.31	1178	MS,RI,S	Floral, honey-like	0.22±0.03	nd	nd	5400
46	*α*-Terpineol	23.24	1197	MS,RI,S	Pleasant, floral	0.22±0.02	nd	nd	0.86
47	Estragole	23.31	1199	MS,RI	Anise-like	0.24±0.04	0.15±0.0	0.80±0.11	0.00013
48	Citronellol	24.83	1231	MS,RI	Fresh, rose-like	nd	0.43±0.05	nd	0.0465
49	Phenethyl acetate	25.89	1254	MS,RI	Rose-like, honey-like, floral	0.02±0.0	0.27±0.04	nd	249.59
50	Anethole	27.34	1285	MS,RI	Anise-like	0.01±0.0	0.24±0.02	0.75±0.03	0.057
51	Safrole	27.42	1287	MS,RI	Camphor wood-like	nd	nd	0.44±0.09	30
52	Indole	27.56	1290	MS,RI,S	Floral, animal-like	0.01±0.0	44.23±0.37	1.84±1.29	0.0081
53	*δ*-Eiemene	29.46	1333	MS,RI	Spicy, anise-like	0.01±0.0	0.49±0.02	nd	n.f.
54	Methyl anthranilate	29.69	1338	MS,RI	Fruity, concord grape	0.04±0.0	0.31±0.04	0.10±0.02	0.000006
55	*α*-Cubebene	29.98	1345	MS,RI	Spicy, citrus	0.28±0.07	0.20±0.01	0.22±0.01	n.f.
56	Phenethyl propanoate	30.16	1349	MS,RI	Sweet, rose-like, fruity, honey-like, strawberry-like	nd	0.54±0.07	nd	18
57	Eugenol	30.19	1350	MS,RI,S	Sweet, spicy, clove-like, woody	nd	0.43±0.03	nd	0.00061
58	Copaene	31.22	1373	MS,RI	Spicy	0.91±0.15	0.18±0.03	0.22±0.0	0.1
59	*β*-Elemene	31.51	1380	MS,RI	Anise-like, spicy	0.10±0.02	0.91±0.02	2.74±0.19	n.f.
60	3-Methyl-1H-indole	31.60	1382	MS,RI	Animal-like	nd	0.28±0.02	nd	0.00003
61	*β*-Cubebene	31.76	1386	MS,RI	Spicy, citrus	0.43±0.10	nd	nd	n.f.
62	1-Ethenyl-1-methyl-2,4-bis(1-methylethenyl)cyclohexane	31.90	1389	MS,RI	——	2.35±0.19	nd	nd	n.f.
63	Methyleugenol	32.46	1401	MS,RI	Clove-like, anise-like, carnation-like	1.81±0.23	0.44±0.01	5.35±0.34	8500
64	Caryophyllene	33.16	1418	MS,RI	Woody, green, spicy, terpenic	4.17±0.23	0.43±0.03	1.57±0.27	13
65	*β*-Gurjunene	33.48	1426	MS,RI	Smoky (soft)	0.01±0.0	0.45±0.06	nd	n.f.
66	*α*-Bergamotene	33.69	1431	MS,RI	Lemon-like, citrus	0.41±0.06	nd	0.14±0.02	n.f.
67	*β*-Phenylethyl butyrate	33.98	1438	MS,RI	Sweet, floral	nd	0.21±0.0	nd	87
68	*epi*-Bicyclosesquiphellandrene	34.14	1442	MS,RI	Spicy, green, celery-like	nd	0.14±0.01	nd	n.f.
69	*trans*-Isoeugenol	34.27	1445	MS,RI,S	Clove-like, woody, spicy, sweet	nd	0.30±0.02	nd	0.006
70	*cis*-Muurola-3,5-diene	34.30	1446	MS,RI	Floral, fragrance	0.12±0.01	nd	nd	n.f.
71	(*E*)-*β*-Famesene	34.55	1452	MS,RI	Green, floral, balsam-like	nd	0.60±0.02	nd	87
72	Humulene	34.59	1453	MS,RI	Woody	1.30±0.21	nd	0.71±0.09	160
73	*γ*-Muurolene	34.83	1458	MS,RI	Floral, fragrance, Ylang-like	0.03±0.0	0.10±0.0	nd	n.f.
74	2-Isopropenyl-4a,8-dimethyl-1,2,3,4,4a,5,6,8a-octahydronaphthalene	35.31	1470	MS,RI	——	0.16±0.02	nd	0.03±0.0	n.f.
75	*α*-Elemene	35.40	1472	MS,RI	Anise-like, spicy	0.15±0.01	0.65±0.09	0.19±0.02	n.f.
76	Germacrene D	35.64	1478	MS,RI	Woody, spicy	0.96±0.12	0.34±0.05	0.59±0.0	n.f.
77	*α*-Curcumene	35.66	1478	MS,RI	Spicy, ginger-like	nd	0.31±0.01	0.33±0.05	n.f.
78	*cis*-*β*-Farnesene	35.79	1481	MS,RI,S	Floral, citrus	0.21±0.05	nd	0.10±0.02	87
79	Phenylethyl 2-methylbutanoate	35.86	1483	MS,RI	Rose-like, fruity	nd	4.44±0.03	1.71±0.16	12
80	*β*-Selinene	35.96	1485	MS,RI	Celery-like	0.85±0.17	nd	nd	1
81	2-Isopropyl-5-methyl-9-methylenebicyclo[4.4.0]dec-1-ene	36.06	1488	MS,RI	——	0.11±0.02	nd	nd	n.f.
82	*α*-Zingiberene	36.16	1490	MS,RI	Ginger-like	0.11±0.03	nd	nd	n.f.
83	*α*-Selinene	36.25	1493	MS,RI	Celery-like	0.70±0.15	0.53±0.12	1.02±0.14	1
84	Methylisoeugenol	36.34	1495	MS,RI	Eugenol-like, sweet, spicy, woody	0.41±0.05	1.39±0.18	11.76±0.88	1600
85	*α*-Guaiene	36.45	1497	MS,RI	Earthy, spicy	nd	0.22±0.01	nd	n.f.
86	*α*-Bulnesene	36.51	1499	MS,RI	Herbal, spicy, green	0.18±0.02	nd	0.27±0.01	n.f.
87	*α*-Farnesene	36.67	1503	MS,RI,S	Woody, green, floral, herbal	nd	0.12±0.02	0.51±0.01	87
88	Eremophilene	36.74	1505	MS,RI	——	1.33±0.07	nd	nd	n.f.
89	*β*-Bisabolene	36.80	1506	MS,RI	Woody, citrus, floral, fruity, green, balsam	0.29±0.08	0.98±0.06	1.07±0.01	n.f.
90	*γ*-Cadinene	36.93	1509	MS,RI	Fruity, sweet, soury, woody, syrup-like, strawberry-like	0.04±0.0	0.74±0.05	0.14±0.01	n.f.
91	*β*-Cadinene	37.05	1512	MS,RI	Minty, camphoraceous, herbaceous, woody, phenolic, warm	0.04±0.0	0.57±0.02	nd	n.f.
92	*δ*-Cadinene	37.19	1516	MS,RI	Herbal, woody	1.06±0.26	nd	1.52±0.14	0.222
93	*trans*-calamenene	37.25	1517	MS,RI	Herbal, spicy	nd	0.71±0.11	nd	2
94	*cis*-Calamenene	37.28	1518	MS,RI	Citrus (faint), green	0.05±0.0	1.00±0.05	0.18±0.03	2
95	*β*-Sesquiphellandrene	37.41	1521	MS,RI	Celery-like	0.03±0.0	0.18±0.01	0.09±0.01	500
96	1,2,3,4,4a,7-Hexahydro-1,6-dimethyl-4-(1-methylethyl)naphthalene	37.71	1529	MS,RI	——	0.05±0.0	0.14±0.01	0.14±0.01	2
97	*α*-Cadinene	37.84	1532	MS,RI	Minty, camphoraceous, herbaceous, woody, phenolic, warm	nd	0.35±0.02	nd	n.f.
98	*α*-Calacorene	38.04	1537	MS,RI	Woody	0.04±0.0	0.97±0.11	0.55±0.07	n.f.
99	Elemicine	38.38	1546	MS,RI	Spicy	0.04±0.01	0.08±0.	0.11±0.0	10000
100	Cadala-1(10),3,8-triene	38.84	1558	MS,RI	Fresh, woody	nd	0.13±0.02	nd	n.f.
101	Nerolidol	38.93	1560	MS,RI,S	Floral, green, citrus, woody, waxy	0.15±0.03	nd	0.55±0.05	10000
102	1,6,7-Trimethylnaphthalene	39.30	1569	MS,RI	Aromatic	nd	0.21±0.03	nd	2.5
103	Hexyl benzoate	39.58	1577	MS,RI	Fresh, balsam, sappy	nd	0.10±0.02	nd	0.0006
104	Caryophyllene oxide	39.62	1577	MS,RI	Woody	0.25±0.02	nd	0.35±0.04	410
105	2-Phenylethyl tiglate	39.74	1581	MS,RI	Berry, caramel-like, floral, tropical, fruity, sweet	nd	0.40±0.07	nd	65
106	Methyl jasmonate	41.99	1640	MS,RI,S	Jasmine-like, floral	nd	0.40±0.09	nd	5700
107	*epi*-*α*-Muurolol	42.02	1640	MS,RI	Floral, sweet	0.09±0.01	nd	0.49±0.04	200000
108	(*Z*)-4-Hexadecen-6-yne	42.66	1658	MS,RI	——	nd	nd	0.28±0.06	n.f.
109	Decahydro-5-methylene-8-vinyl-2-naphthalenemethanol	42.97	1666	MS,RI	——	nd	nd	0.41±0.04	n.f.
110	Cadalene	43.04	1668	MS,RI	Coal tar-like	nd	0.09±0.02	0.37±0.01	10
111	(*Z,E*)-Farnesol	44.00	1694	MS,RI	Limette-like	nd	0.17±0.0	nd	1000
112	Heptadecane	44.23	1700	MS,RI,S	Alkane-like	nd	0.16±0.01	nd	10000000
113	Methyl tetradecanoate	45.10	1724	MS,RI	Fatty, waxy	nd	0.12±0.01	nd	0.5
114	Nootkatone	46.53	1764	MS,RI	Citrus, grapefruit-like	nd	nd	0.54±0.01	280
115	Octadecane	47.80	1800	MS,RI,S	Alkane-like	nd	0.23±0.04	nd	0.02
116	9-Nonadecene	50.42	1877	MS,RI	Alkane-like	nd	0.53±0.06	nd	n.f.
117	Nonadecane	51.21	1900	MS,RI,S	Alkane-like	0.01±0.0	3.09±0.18	nd	10000000
118	Methyl hexadecanoate	52.02	1924	MS,RI	Oily, waxy, fatty	Trace	4.60±0.23	nd	>2000
119	*n*-Hexadecanoic acid	53.21	1958	MS,RI	Waxy, creamy, candle-like	Trace	nd	0.37±0.02	>10000
120	Ethyl hexadecanoate	54.46	1994	MS,RI	Oily, waxy, fatty	0.03±0.0	0.05±0.01	4.71±0.14	2000
121	*E*-15-Heptadecenal	56.66	2081	MS,RI	Grassy, green, pungent smelly	nd	0.12±0.0	nd	n.f.
122	Methyl linoleate	56.93	2092	MS,RI	Oily, fatty, woody	Trace	2.98±0.25	0.72±0.05	450
123	Methyl linolenate	57.03	2096	MS,RI	Fatty, waxy	nd	1.54±0.16	nd	450
124	Heneicosane	57.14	2100	MS,RI	Alkane-like	0.01±0.0	0.93±0.10	nd	10000000
125	Methyl (*Z,Z*)-9,15-octadecadienoate	57.90	2144	MS,RI	Fatty, waxy	nd	0.19±0.0	nd	450
126	Ethyl linoleate	58.15	2159	MS,RI	Oily, fatty, woody	0.11±0.11	nd	3.25±0.28	450
127	Ethyl linolenate	58.25	2164	MS,RI	Fatty, waxy	0.07±0.01	nd	2.66±0.44	450
128	Tricosane	60.84	2301	MS,RI,S	Alkane-like	0.01±0.0	0.20±0.01	0.24±0.03	10000000

Each value in the table was presented as the mean ± standard deviation (n = 3).

ζ Identification method. MS, identification based on the NIST 2017 mass spectral database; RI, retention index; S, the compounds were identified using authentic standard compounds.

ψ Odor description found in the literature (Flavornet; The LRI and Odour Database).

‘——‘, no odor description information was found in the literature.

# All the odor thresholds were obtained from: L. J. van Gemert, Compilations of Odour Threshold Values in Air, Water and Other Media, 2nd ed. (Utrecht: Oliemans Punter & Partners BV, 2011).

nd, not detectable.

‘n.f.’, data was not found in the literature.

**Figure 2:**
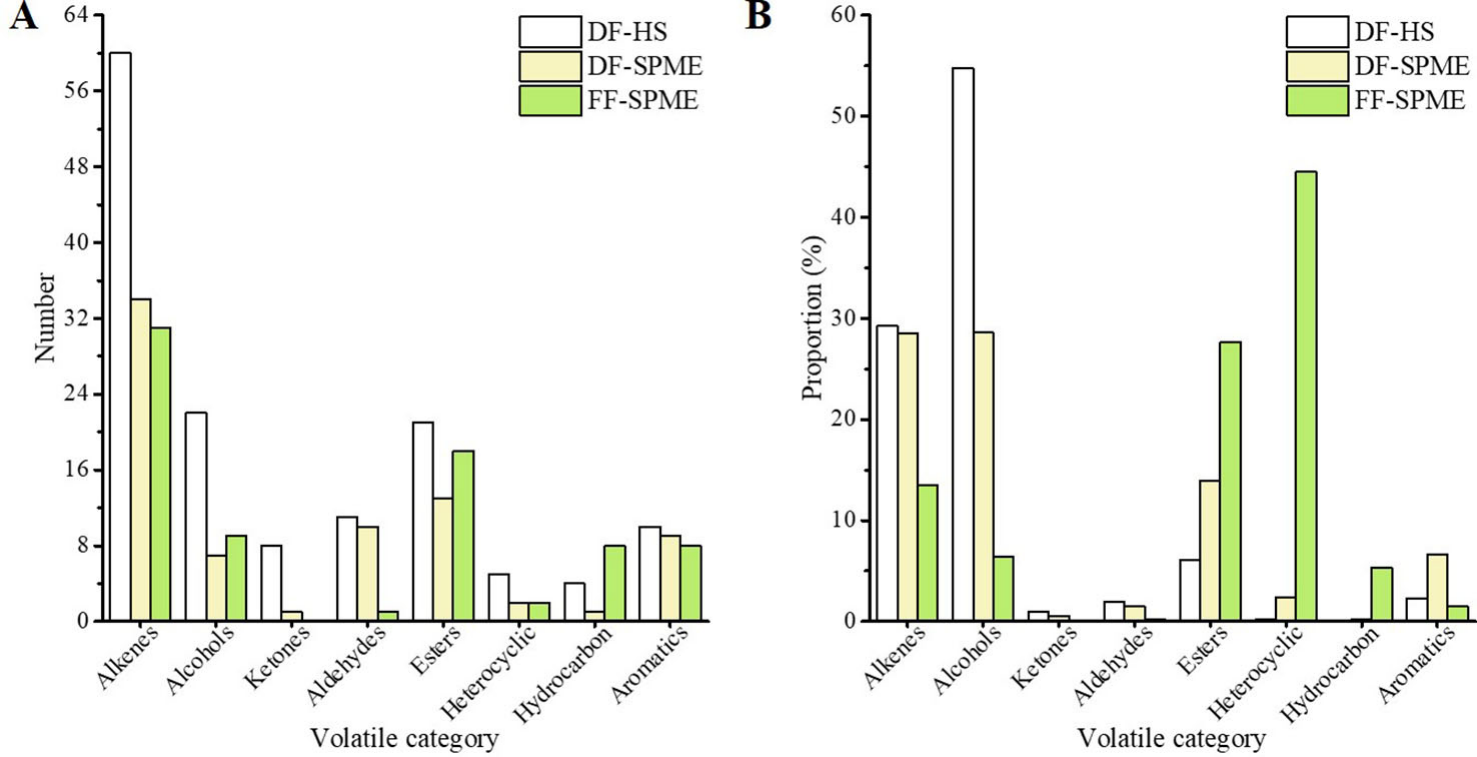
The number **(A)** and proportion **(B)** of the volatile categories in white champaca from fresh and dried flowers.

The identified compounds comprised alkenes, alcohols, ketones, aldehydes, esters, heterocyclics, hydrocarbons, and aromatics. Alkene compounds dominated the volatiles in terms of numbers; 31, 34, and 60 volatiles were found in FF-SPME, DF-SPME, and DF-HS, respectively, followed by ester and alcohol compounds (**Figure 2A**). Dried flowers contained higher numbers of aldehydes, aromatics, and heterocyclic compounds, while the highest number of hydrocarbon compounds was found in fresh flowers. Ketone compound was absent in FF-SPME but reached a maximum of eight compounds in DF-HS. Significant differences were observed in proportions of these volatile categories among the three samples (**Figure 2B**). Alkene and alcohol compounds dominated the volatiles in dried flowers, with the sum of the two categories comprising more than 57.0% and 84.0% of the total amounts in DF-SPME and DF-HS, respectively. Conversely, fresh flowers were characterized by higher proportions of heterocyclics (44.51%), esters (27.67%), and hydrocarbon compounds (5.34%), while showing the lowest proportions of alkenes, alcohols, aldehydes, and aromatics.

#### 3.1.1 Volatile profiling of FF-SPME

Among the 78 identified volatiles in fresh flowers, indole was the most abundant (44.23%), which was not in agreement with the findings of a previous study showing that linalool was the most abundant in white champaca (Xia et al., 2010), which might be attributed to the different detection method used. Indole occurs naturally in many aromatic plants and is also considered to be the key volatile compound contributing to the floral odor in tea (Guo et al., 2025a). Other abundant compounds (>3.0%) included methyl hexadecanoate, methyl benzoate, phenylethyl 2-methylbutanoate, and phenylethyl alcohol. The first one exhibiting oily or fatty odors was also identified in tea aroma (Guo et al., 2021a); methyl benzoate having floral or fruity odors was the compound with the second highest content in fresh flowers of white champaca, and phenylethyl 2-methylbutanoate contributes to rose-like or fruity odors reported in natural food products (Barros-Castillo et al., 2022). The latter three compounds, together with indole, might contribute to the floral flavor of white champaca flowers. Phenylethyl alcohol possessing a rose-like odor is a known aroma-active compound in yellow tea and oolong tea (Shi et al., 2021; Guo et al., 2022).

Similar to the cases of methyl hexadecanoate, methyl linoleate and methyl linolenate imparting oily or fatty odors were found in fresh flowers with a high relative amount (2.98% and 1.54%, respectively), and the former one is also identified in the flower concrete of *Michelia champaca* (Kaiser, 1991). Additionally, methyl 2, 3-dimethylbutanoate, methylisoeugenol, *cis*-calamenene, and linalool were also abundant with relative amounts above 1.0%. Methylisoeugenol exhibits eugenol-like odor, *cis*-calamenene has citrus or green notes, and linalool imparting floral flavor is considered to be the key volatile compound in aromatic plants (Guo et al., 2021b; Guo et al., 2021c; Zhang et al., 2021). Up to eight linear hydrocarbon compounds were identified in FF-SPME, in which nonadecane was abundant with a relative amount of 3.09%, which might have a certain impact on the presentation of overall aroma characteristics. In addition, benzyl nitrile and methyl anthranilate having almond-like and fruity odors, respectively, were found not only in FF-SPME with a low relative amount but also in jasmine flowers (Issa et al., 2020). Both estragole and anethole exhibiting anise-like odor were found in FF-SPME with relative amounts lower than 0.25%, which have been previously identified in various edible aromatic plants and jasmine flowers (Issa et al., 2020; Dief et al., 2025). Eugenol contributes to the clove-like and sweet odors identified in the flower concrete of *M. champaca* L (Kaiser, 1991).; (*E*)-*β*-famesene having floral or green odors (Issa et al., 2020) and *epi*-bicyclosesquiphellandrene and *α*-cadinene with spicy and minty odors (Kamli et al., 2024), respectively, were also found in FF-SPME with relatively low amounts.

#### 3.1.2 Volatile profiling of DF-SPME

Significant compositional differences were observed between FF-SPME and DF-SPME. DF-SPME was characterized by a marked increase in alkene and alcohol compounds, specifically linalool, methylisoeugenol, and methyleugenol, which accounted for 43.40% of the total amount. The contents of these volatiles were 8.46–18.91 times of those in FF-SPME. Linalool with a floral odor had the highest content in dried flowers of white champaca, consistent with the results from Xia et al (Xia et al., 2010). Methyleugenol imparting clove-like or anise-like odors is a flavoring ingredient commonly found in natural plants (Fukushima et al., 2020). Although the content of indole in dried flowers was higher than 1.0%, it was only 4.16% of that in FF-SPME. Similarly, phenylethyl alcohol, phenylethyl 2-methylbutanoate, methyl benzoate, and methyl linoleate were significantly lower in DF-SPME than in FF-SPME. Conversely, *α*-ocimene, *β*-elemene, caryophyllene, *α*-selinene, *β*-bisabolene, and ethyl hexadecanoate were dramatically enhanced. *α*-Ocimene and *β*-bisabolene having woody odors, together with *β*-elemene and *α*-selinene with anise-like and green notes, respectively, are naturally identified in essential oils of aromatic plants (Kamli et al., 2024). Caryophyllene having woody or spicy odors is the main volatile compound in essential oil of white champaca (Suhem et al., 2017). The latter one with oily and fatty odors was identified in DF-SPME with a relatively high amount of 4.71%.

Meanwhile, some volatile compounds in DF-SPME with amounts higher than 1.0% were not detected in FF-SPME, such as 2-mthylbutanoic acid, *δ*-cadinene, ethyl linoleate, and ethyl linolenate. The latter two compounds having fatty and oily odors are commonly found in teas (Guo et al., 2022); *δ*-cadinene with herbal and woody odors is also a volatile compound in jasmine flowers (Issa et al., 2020). The odor characteristic of 2-methylbutanoic acid is related to the concentration, the high level of 2-methylbutanoic acid yields an unpleasant goat cheese-like aroma with a pungent flavor, while a low amount of 2-methylbutanoic acid is associated with a fruity odor. It is also the main volatile compound of white champaca extract (Samakradhamrongthai et al., 2016). In comparison to FF-SPME, some green flavor compounds [hexanal and (*E,E*)-2,4-hexadienal] were newly found in DF-SPME. Hexanal and (*E, E*)-2,4-hexadienal with grassy and odors, respectively, are the major volatile compounds in tea aroma (Guo et al., 2021c; Guo et al., 2025a). Furthermore, the volatiles, benzaldehyde, benzene acetaldehyde, linalool oxide I (*cis*, furanoid), linalool oxide (pyranoid), nerolidol, etc., are regarded as the key volatile compounds conveying floral flavor for tea aroma (Guo et al., 2025a) were not detected in FF-SPME, might be related to the different states of white champaca flowers. Ethyl acetate (fruity odor), (*E*)-2-butenal (pungent flavor), safrole (camphor wood-like odor), and vanillin (vanilla-like, milky odors) were only identified in dried flower aroma extracted using the HS method.

#### 3.1.3 Volatile profiling of DF-HS

HS extraction identified 150 volatile compounds in dried flowers, representing 1.92 and 1.81 times the number found in FF-SPME and DF-SPME, respectively. Only 13 volatiles had relative amounts greater than 1.0%, with linalool being the most abundant, accounting for 52.10% of the total amount, confirming its status as the signature compound in dried white champaca. Similar to DF-SPME, 2-methylbutanoic acid, *δ*-cadinene, methyleugenol, *α*-ocimene, and caryophyllene were abundant, and the contents of the first three were significantly lower than those in DF-SPME, while the latter two showed opposite trends. Notably, volatiles unique to DF-HS (ethyl 2-methylpropanoate, eremophilene, etc.), or absent in fresh flowers (ethyl 2-methylbutanoate, eucalyptol, *trans*-*β*-ocimene, and humulene), had high relative amounts larger than 1.0% in dried flower aroma extracted using the HS method. Ethyl 2-methylpropanoate and ethyl 2-methylbutanoate significantly contribute to the fruity odor; eucalyptol with camphor-like or green notes provides the characteristic aroma of the *Allium tenuissimum* L. flower (Zhang et al., 2021). Humulene is a volatile ingredient in spice plants providing woody aroma (Guo et al., 2022), and *trans*-*β*-ocimene is commonly found in teas offering a strong impression of herbal or floral flavor (Guo et al., 2021b).

In addition, there were some low-molecular-weight volatile compounds such as butanal, 2,3-butanedione, 2-methylfuran, 2-ethylfuran, 3-methylbutanal, 2-methylbutanal, ethyl propanoate, methyl 2-methylbutanoate, 3-methyl-2-butenal, furfural, and 2-heptanone that were solely identified in DF-HS with a relatively low amount. Methyl 2-methylbutanoate is considered to be the characteristic volatile compound in white champaca with a fruity flavor (Ueyama and Hashimoto, 1992). Similarly, 2-methylbutanal, 3-methylbutanal, ethyl propanoate, and 2-heptanone were important contributors to the fruity note, offering fruity, apple-like, pineapple-like, and pear-like odors, respectively, which are regarded as the aroma-active compounds in teas or vinegar (Zhang et al., 2021; Liu et al., 2022; Ding et al., 2026). The nutty or caramel-like flavor compounds 2-methylfuran and 2-ethylfuran are the Maillard reaction volatiles, which have been reported in foods with thermal treatment (Cao et al., 2022). 2, 3-Butanedione (trace amount) and butanal with butter-like and green odors, respectively, have been characterized as key odorants in oolong tea infusion based on determined aroma intensity (Ding et al., 2026). It has been reported that 3-methyl-2-butenal (trace amount) is widely identified in tea aroma contributing to burnt or cocoa-like odors (Guo et al., 2021a; Guo et al., 2021b; Guo et al., 2022). Furfural with caramel-like, almond-like, or sweet odors can be derived from serine by Strecker degradation during heating (Huang et al., 2023). Furthermore, pentanoic acid, 2-methylhexanoic acid, ethyl 2-methyl-2-butenoate, and hexanoic acid, having smelly, sour, mushroom-like, and acrid flavor, respectively, which were not appropriate for the aroma of white champaca flowers, were only identified in DF-HS. Instead, methyl benzoate, ethyl benzoate, phenylethyl 2-methylbutanoate, and *α*-farnesene were not detected in DF-HS. *α*-Farnesene has been identified as an important volatile compound contributing to the woody, floral, or green odors for teas (Guo et al., 2021a; Guo et al., 2021b). Although methylisoeugenol, indole, and phenylethyl alcohol were identified in all three samples, their contents were significantly lower in DF-HS than those in FF-SPME and DF-SPME. Moreover, linalool oxide II exhibiting floral odor was only identified in DF-HS.

The distinct aroma profiles between fresh and dried flowers are likely linked to the fundamental role of water as a solvent and matrix. Its removal during drying may concentrate non-polar volatiles and possibly trigger reactions that produce new compounds. However, the specific biochemical pathways driving these compositional shifts remain unclear and warrant deeper investigation.

### 3.2 Differential volatile profiles of white champaca from fresh and dried flowers

**Figure 3:**
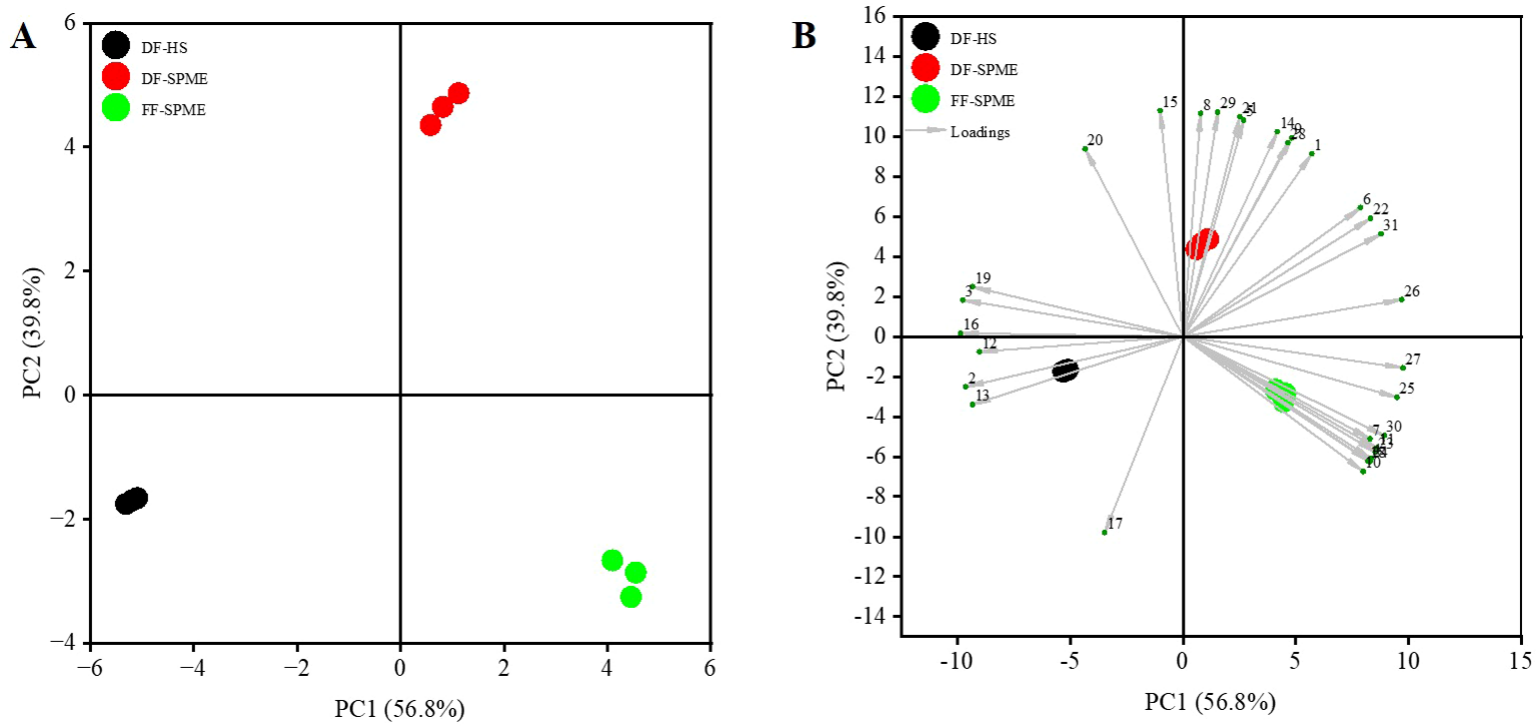
PCA of white champaca from fresh and dried flowers. **(A)** The score plot of PCA. **(B)** The biplot of PCA. Compound numbers correspond to **Table 2**.

PCA was performed to visualize the discrimination among samples based on the common volatiles (**Table 2**). The first two principal components (PC 1 and PC 2) explained 56.8% and 39.8% of the total variance, respectively, with a cumulative contribution of 96.6%. This high cumulative variance indicates that the model effectively captures the majority of the information in the dataset. As shown in **Figure 3A**, the three samples were well separated, which were distributed in different regions, indicating that the significant differences on volatile properties were observed among the three samples. The clear segregation validates that the drying process and extraction technique induce systematic changes in aroma composition. The distance between DF-SPME and DF-HS along the PC1 axis suggests that the extraction method significantly influences the captured volatile profile, likely due to HS sensitivity to low-boiling-point compounds.

**Table 2:** The relative amounts of common volatiles in white champaca from fresh and dried flowers.

No.	Volatile compounds	Relative amount (%)		
		DF-HS	FF-SPME	DF-SPME
1	Benzaldehyde	0.10±0.02	0.24±0.03	0.49±0.02
2	*α*-Ocimene	4.33±0.39	0.37±0.04	1.02±0.01
3	Linalool	52.10±2.14	1.39±0.30	26.29±0.26
4	Phenylethyl alcohol	0.67±0.05	3.08±0.10	1.02±0.04
5	(*4E*,*6Z*)-*allo*-Ocimene	0.04±0.01	0.08±0.02	0.18±0.01
6	Cosmene	0.03±0.0	0.26±0.04	0.33±0.05
7	Benzyl nitrile	0.03±0.0	0.38±0.08	0.07±0.01
8	Estragole	0.24±0.04	0.15±0.0	0.80±0.11
9	Anethole	0.01±0.0	0.24±0.02	0.75±0.03
10	Indole	0.01±0.0	44.23±0.37	1.84±1.29
11	Methyl anthranilate	0.04±0.0	0.31±0.04	0.10±0.02
12	*α*-Cubebene	0.28±0.07	0.20±0.01	0.22±0.01
13	Copaene	0.91±0.15	0.18±0.03	0.22±0.0
14	*β*-Elemene	0.10±0.02	0.91±0.02	2.74±0.19
15	Methyleugenol	1.81±0.23	0.44±0.01	5.35±0.34
16	Caryophyllene	4.17±0.23	0.43±0.03	1.57±0.27
17	*β*-copaene	0.06±0.01	0.07±0.0	0.05±0.0
18	*α*-Elemene	0.15±0.01	0.65±0.09	0.19±0.02
19	Germacrene D	0.96±0.12	0.34±0.05	0.59±0.0
20	*α*-Selinene	0.70±0.15	0.53±0.12	1.02±0.14
21	Methylisoeugenol	0.41±0.05	1.39±0.18	11.76±0.88
22	*β*-Bisabolene	0.29±0.08	0.98±0.06	1.07±0.01
23	*γ*-Cadinene	0.04±0.0	0.74±0.05	0.14±0.01
24	*cis*-Calamenene	0.05±0.0	1.00±0.05	0.18±0.03
25	*β*-Sesquiphellandrene	0.03±0.0	0.18±0.01	0.09±0.01
26	1,2,3,4,4a,7-Hexahydro-1,6-dimethyl-4-(1-methylethyl)naphthalene	0.05±0.0	0.14±0.01	0.14±0.01
27	*α*-Calacorene	0.04±0.0	0.97±0.11	0.55±0.07
28	Elemicine	0.04±0.01	0.08±0.	0.11±0.0
29	Ethyl hexadecanoate	0.03±0.0	0.05±0.01	4.71±0.14
30	Methyl linoleate	Trace	2.98±0.25	0.72±0.05
31	Tricosane	0.01±0.0	0.20±0.01	0.24±0.03

As shown in **Figure 3B**, FF-SPME exhibited high scores in positive PC 1 and negative PC 2 where the loadings of volatile compounds including methyl linoleate, *α*-calacorene, *β*-sesquiphellandrene, and indole were most abundant in fresh flowers of white champaca. For the dried flowers, DF-SPME had high scores in positive PC 1 and PC 2, which contained high loadings of estragole, ethyl hexadecanoate, methylisoeugenol, (4*E*,6*Z*)-*allo*-ocimene, *β*-elemene, etc., the characteristic volatile compounds with fruity, floral, or spicy odors in dried flower aroma obtained via SPME extraction. DF-HS had high scores in negative PC 1 and PC 2 with six abundant characteristic volatile compounds, namely, germacrene D, *β*-copaene, linalool, *α*-cubebene, *α*-ocimene, and copaene, among which linalool was the most characteristic volatile compound, consistent with the results from Xia et al. (2010) that linalool was a major volatile compound in white champaca flower.

### 3.3 Odor profiles of white champaca from fresh and dried flowers

The contribution of each volatile compound to the overall aroma was evaluated using rOAV, which considers both concentration and odor threshold (Zhu et al., 2020). Components with rOAV greater than 1 are considered as the key aroma odorant of the sample, while those with 0.1 ≤ rOAV < 1 are important modifiers (Xiao et al., 2022). A total of 23 volatiles had rOAV values higher than 0.1 among the three samples, including 12 volatile compounds with rOAV values greater than 1, which are shown in **Table 3**; detailed information on rOAV values of the identified volatiles is provided in **Supplementary Table 2**. As for fresh flowers of white champaca, 10 volatiles were identified with rOAV above 0.1, and some volatiles, such as (4*E*, 6*Z*)-*allo*-ocimene and *α*-ocimene, had the characteristic green flavor. In DF-SPME, 13 volatiles were identified with an rOAV higher than 0.1. 2-Methylbutanoic acid, (*E, E*)-2, 4-hexadienal, *trans*-*β*-ocimene, (*E,E*)-2,4-decadienal, and vanillin, among others, were newly found in DF-SPME in comparison to FF-SPME, while hexyl benzoate was not observed in DF-SPME. A total of 13 volatile constituents were identified with an rOAV higher than 0.1 in DF-HS, among which acetic acid, 2-heptanone, and *o*-cymene were uniquely found in dried flower aroma extracted using the HS method.

**Table 3:** The volatiles with rOAV higher than 0.1 in white champaca from fresh and dried flowers.

Volatile compounds	rOAV (%)		
	DF-HS	FF-SPME	DF-SPME
Acetic acid	0.14	nd	nd
3-Methylbutanal	4.43	nd	nd
Ethyl 2-methylpropanoate	88.98	nd	nd
2-Methylbutanoic acid	0.24	nd	0.78
2-Heptanone	0.83	nd	nd
(*E,E*)-2,4-Hexadienal	nd	nd	0.65
Pentanoic acid	2.84	nd	nd
*o*-Cymene	0.16	nd	nd
*trans*-*β*-Ocimene	0.78	nd	0.10
*α*-Ocimene	1.07	0.04	0.33
Methyl benzoate	nd	12.13	0.46
Linalool	100.00	1.12	65.58
Phenylethyl Alcohol	0.15	0.28	0.29
(4*E*,6*Z*)-*allo*-Ocimene	0.02	0.02	0.11
Ethyl benzoate	nd	2.22	3.47
Estragole	8.43	2.22	36.77
Indole	0.01	10.60	1.36
(*E,E*)-2,4-Decadienal	0.01	nd	0.12
Methyl anthranilate	29.48	100.00	100.00
Eugenol	nd	1.38	nd
3-Methyl-1H-indole	nd	18.29	nd
Vanillin	nd	nd	0.49
Hexyl benzoate	nd	0.34	nd

nd, not detectable.

**Figure 4:**
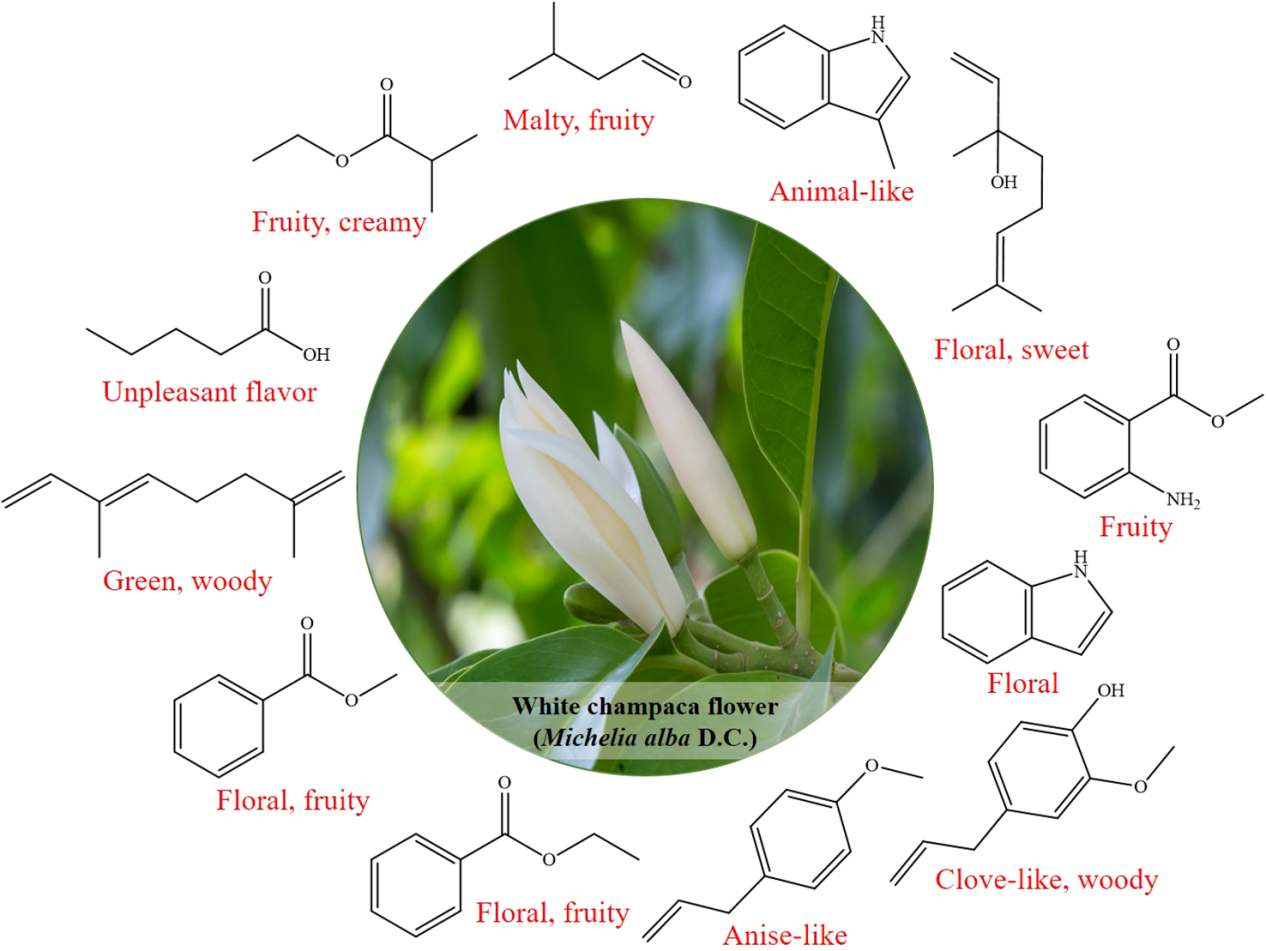
The potential key volatile compounds with rOAV above one in white champaca from fresh and dried flowers.

Moreover, methyl anthranilate had the largest rOAV value (rOAV = 100) in both FF-SPME and DF-SPME samples, indicating that it had the greatest contribution to the fruity odor of the two samples (**Figure 4**). Methyl benzoate and ethyl benzoate with rOAV values of 12.13 and 2.22, respectively, were positive contributors to the fruity odor in FF-SPME. The floral odor might be attributed to linalool, indole, eugenol, and 3-methyl-1H-indole, owing to their rOAV values being above 1, which were 1.12, 10.60, 1.38, and 18.29, respectively. In addition to methyl anthranilate, the other four volatiles, namely, linalool, ethyl benzoate, estragole, and indole, were commonly identified in DF-SPME with rOAV higher than 1. Linalool with the second highest rOAV value of 65.58, together with indole (rOAV = 1.36), might be the key volatile compounds providing floral odor in DF-SPME, and estragole (rOAV = 36.77) might have greatly contributed to the spicy note in dried flowers of white champaca. Different from the odor characteristics of white champaca aroma extracted using the SPME method, linalool with the highest rOAV value of 100 had the greatest contribution to the floral odor in DF-HS. Ethyl 2-methylpropanoate (rOAV = 88.98) and methyl anthranilate (rOAV = 29.48) were major contributors to the fruity odor in DF-HS. In addition, 3-methylbutanal was only identified in DF-HS with an rOAV value of 4.43, which had a positive contribution to the fruity scent for dried flowers obtained using HS extraction.

It is important to note that while rOAV predicts sensory impact based on chemical concentration and threshold, it does not fully replicate human sensory perception. Interactions such as synergy or masking between compounds are not captured by rOAV alone. Therefore, while we identify potential key odorants, future sensory evaluation panels are required to confirm the actual perceived aroma quality. From a practical perspective, the dominance of linalool in dried flowers suggests that they are highly suitable for the perfume industry, where linalool is prized for its fresh, floral scent.

## 4 Conclusions

This study provides a comprehensive comparative analysis of the volatile profiles of white champaca flowers in fresh and dried states using SPME and HS extraction methods coupled with GC-MS. The results demonstrate that the physical state of the flower and the extraction technique significantly influence the observed aroma profile. PCA revealed distinct clustering among the samples, confirming substantial differences in volatile composition induced by drying and extraction methods. rOAV analysis identified 15 key odorants (rOAV > 1) contributing to the overall aroma. Notably, the aroma characteristics were dominated by different compounds depending on the sample state: methyl anthranilate (rOAV = 100) was the primary contributor to fruity notes in fresh flowers and SPME-dried samples, whereas linalool (rOAV = 100) emerged as the dominant compound imparting floral scent in dried flowers, particularly in the HS extract. This study relied on rOAV calculations and chromatographic data to infer sensory properties. While these chemical metrics provide strong indicators of key odorants, they do not fully replicate human sensory perception. Therefore, future research should incorporate sensory evaluation panels to validate the actual aroma perception and investigate potential synergistic effects. Additionally, the specific mechanisms behind the release of bound volatiles during drying warrant further investigation using isotope labeling or enzymatic analysis. These findings offer a valuable chemical basis for optimizing the processing and utilization of white champaca flowers in the tea, cosmetic, and aromatherapy industries.

## Data Availability

The original contributions presented in the study are included in the article/**Supplementary Material**. Further inquiries can be directed to the corresponding author.
