# Supplementary material for: Analysis of volatile compounds emitted from white champaca flowers (Michelia alba D.C.) via HS-GC-MS and SPME-GC-MS technology
Source: Front Plant Sci. 2026 Apr 1;17:1780030. doi: 10.3389/fpls.2026.1780030 (PMC13079565; doi:10.3389/fpls.2026.1780030)
Supplement: Supplementary file 1 [file Table1.docx]

**Supplementary material**

**Analysis of volatile compounds emitted from white champaca flowers (*Michelia alba* D. C.) by HS-GC-MS and SPME-GC-MS technology**

(Abbreviated running title: Aroma profiles in white champaca flowers)

Xiangyang Guo^a,b,*^, Beijun Cao^a^

*^a^ College of Tea and Food Science, Xinyang Normal University, Xinyang 464000, China*

*^b^ College of Chemistry and Environmental Engineering, Shenzhen University, Shenzhen 518060, China*

*** Corresponding author:**

Xiangyang Guo, College of Chemistry and Environmental Engineering, Shenzhen University, 3688 Nanhai Ave, Shenzhen 518060, China; E-mail address: xiangyang.guo@ahau.edu.cn

**Table S1** The relative amounts, odor description, and odor threshold value of identified volatiles in white champaca from fresh and dried flowers

| No. | Volatile compounds | RT (min) | RI | ID ^ζ^ | Odor quality ^ψ^ | Relative amount (%) | | | Threshold (mg/m^3^) |
| --- | --- | --- | --- | --- | --- | --- | --- | --- | --- |
|  |  |  |  |  |  | DF-HS | FF-SPME | DF-SPME |  |
| 1 | Butanal | 2.05 | 576 | MS,RI | Green, musty, pungent | 0.12 | nd | nd | 0.1 |
| 2 | 2,3-Butanedione | 2.20 | 592 | MS,RI | Butter-like | 0.00 | nd | nd | 0.00018 |
| 3 | Acetic acid | 2.23 | 595 | MS,RI | Sour | 0.40 | nd | nd | 0.013 |
| 4 | Ethyl Acetate | 2.36 | 608 | MS,RI | Fruity | nd | nd | 0.03 | 0.88 |
| 5 | 2-Methylfuran | 2.42 | 615 | MS,RI | Nutty, sweet, musty | 0.03 | nd | nd | 200 |
| 6 | (*E*)-2-Butenal | 2.71 | 644 | MS,RI | Pungent | nd | nd | 0.02 | 0.067 |
| 7 | 3-Methylbutanal | 2.79 | 653 | MS,RI | Malty, fruity, apple/peach-like | 0.34 | nd | nd | 0.00035 |
| 8 | 2-Methylbutanal | 2.96 | 670 | MS,RI | Pungent, coffee/cocoa-like, fruity | 0.98 | nd | nd | 0.1 |
| 9 | 2-Ethylfuran | 3.33 | 703 | MS,RI | Butter, caramel-like, burnt | 0.02 | nd | nd | 8000 |
| 10 | Ethyl propanoate | 3.52 | 712 | MS,RI | Fruity, pineapple-like | 0.07 | nd | nd | 40.3 |
| 11 | 1-Methoxypentane | 4.12 | 739 | MS,RI | —— | 0.09 | nd | nd | n.f. |
| 12 | Ethyl 2-methylpropanoate | 4.51 | 756 | MS,RI | Fruity, creamy | 2.12 | nd | nd | 0.00011 |
| 13 | Toluene | 4.67 | 763 | MS,RI,S | Sweet, aromatic | 0.02 | nd | nd | 0.3 |
| 14 | Methyl 2-methylbutanoate | 4.93 | 775 | MS,RI | Fruity (pungent), fatty, creamy, green | 0.34 | nd | nd | 8.76 |
| 15 | 3-Methyl-2-butenal | 5.12 | 784 | MS,RI | Burnt, cocoa-like | 0.00 | nd | nd | 500 |
| 16 | Hexanal | 5.60 | 803 | MS,RI,S | Grassy, green, fresh, fatty | 0.30 | nd | 0.15 | 0.23 |
| 17 | Methylpent-4-enylamine | 6.03 | 815 | MS,RI | —— | nd | 0.08 | nd | n.f. |
| 18 | Methyl 2,3-dimethylbutanoate | 6.29 | 822 | MS,RI | —— | nd | 1.37 | nd | n.f. |
| 19 | Furfural | 6.40 | 825 | MS,RI,S | Sweet, bready, caramel | 0.09 | nd | nd | 2.8 |
| 20 | Methyl valerate | 6.60 | 831 | MS,RI | Fruity, sweet | 0.01 | nd | nd | 20 |
| 21 | Ethyl 2-methylbutanoate | 7.23 | 848 | MS,RI | Fruity, peely (apple, pineapple) | 2.66 | nd | 0.14 | 11.7 |
| 22 | 2-Hexenal | 7.27 | 849 | MS,RI,S | Grassy, herbal | nd | nd | 0.25 | 0.48 |
| 23 | 2-Methylbutanoic acid | 7.78 | 863 | MS,RI | Pungent, goat cheese-like, fruity | 1.04 | nd | 2.60 | 0.02 |
| 24 | Methyl tiglate | 8.66 | 888 | MS,RI | Aromatic | nd | 0.09 | nd | 130 |
| 25 | 2-Heptanone | 8.74 | 890 | MS,RI | Pear-like, fruity | 0.63 | nd | nd | 0.0035 |
| 26 | (*E,E*)-2,4-Hexadienal | 9.57 | 910 | MS,RI,S | Green, sweet, fruity | nd | nd | 0.20 | 0.0018 |
| 27 | Pentanoic acid | 9.64 | 912 | MS,RI | Unpleasant flavor, smelly | 0.10 | nd | nd | 0.00016 |
| 28 | 2-Methylhexanoic acid | 9.92 | 918 | MS,RI | Sour, fatty, roasted food-like | 0.29 | nd | nd | 2700 |
| 29 | Butyrolactone | 10.38 | 928 | MS,RI | Acetone-like | 0.28 | nd | nd | ＞1000 |
| 30 | *α*-Pinene | 10.50 | 931 | MS,RI,S | Pine-like | 0.81 | nd | nd | 0.1 |
| 31 | Ethyl 2-methyl-2-butenoate | 10.84 | 938 | MS,RI | Mushroom-like | 0.05 | nd | nd | 65 |
| 32 | Camphene | 11.23 | 947 | MS,RI | Camphor-like | 0.24 | nd | nd | 30 |
| 33 | Benzaldehyde | 11.83 | 960 | MS,RI,S | Almond-like, fruity, cherry-like, powdery | 0.10 | 0.24 | 0.49 | 0.085 |
| 34 | *β*-Thujene | 12.33 | 971 | MS,RI | Woody, spicy, citrus | 0.40 | nd | nd | n.f. |
| 35 | *β*-Pinene | 12.53 | 975 | MS,RI,S | Pine-like | 0.94 | nd | nd | 0.18 |
| 36 | 6-Methyl-5-hepten-2-one | 12.99 | 985 | MS,RI | Fruity, apple-like, citrus | 0.04 | nd | nd | 0.01889 |
| 37 | *β*-Myrcene | 13.18 | 989 | MS,RI,S | Woody, resinous, musty, balsamic, ethereal | 0.27 | nd | 0.12 | 0.1125 |
| 38 | Hexanoic acid | 13.53 | 997 | MS,RI | Acrid flavor | 0.09 | nd | nd | 0.0048 |
| 39 | Ethyl hexanoate | 13.69 | 1000 | MS,RI,S | Green apple | 0.33 | nd | 0.06 | 70 |
| 40 | *α*-Phellandrene | 13.91 | 1005 | MS,RI | Spicy, pepper-like | 0.06 | nd | nd | 3.9 |
| 41 | (*E,E*)-2,4-Heptadienal | 14.21 | 1011 | MS,RI,S | Fatty, green, oily, cinnamon-like | nd | nd | 0.15 | 0.057 |
| 42 | 3-Pyridinecarbonitrile | 14.26 | 1012 | MS,RI | Burnt, cocoa-like | 0.01 | nd | 0.56 | n.f. |
| 43 | *α*-Terpinene | 14.42 | 1015 | MS,RI,S | Citrusy, woody, lemon-like | 0.10 | nd | nd | 7.9 |
| 44 | *o*-Cymene | 14.81 | 1023 | MS,RI | Aromatic | 0.17 | nd | nd | 0.005 |
| 45 | Limonene | 15.02 | 1028 | MS,RI,S | Citrus, lemon, orange,green, etherel | 0.26 | nd | 0.06 | 0.21 |
| 46 | Eucalyptol | 15.20 | 1031 | MS,RI | Camphor-like, herb | 1.76 | nd | 0.16 | 0.15 |
| 47 | *trans*-*β*-Ocimene | 15.51 | 1038 | MS,RI,S | Warm, floral, herbal, sweet | 3.16 | nd | 0.31 | 0.0187 |
| 48 | Benzeneacetaldehyde | 15.79 | 1043 | MS,RI,S | Floral, rose, cherry-like | 0.11 | nd | 0.07 | 6.3 |
| 49 | *α*-Ocimene | 16.04 | 1049 | MS,RI,S | Green, woody, tropical | 4.33 | 0.37 | 1.02 | 0.0187 |
| 50 | *γ*-Terpinene | 16.46 | 1057 | MS,RI,S | Citrus, lemon-like, woody, spicy, juicy | 0.65 | nd | nd | 55.0 |
| 51 | Linalool oxide Ⅱ (trans, furanoid) | 17.31 | 1074 | MS,RI,S | Sweet, floral, creamy | 0.27 | nd | nd | 60 |
| 52 | 1-Octanol | 17.41 | 1076 | MS,RI,S | Green, citrus, fatty, coconut-like | nd | 0.03 | nd | 0.022 |
| 53 | 1-Phenyl-1-butene | 17.68 | 1082 | MS,RI | —— | nd | nd | 0.03 | 2.2 |
| 54 | *α*-Terpinolen | 17.75 | 1084 | MS,RI | Fresh, woody, citrus, pine-like, sweet | 0.11 | nd | nd | 200 |
| 55 | Linalool oxide Ⅰ (cis, furanoid) | 17.88 | 1086 | MS,RI,S | Sweet, floral, creamy | 0.28 | nd | 0.07 | 100 |
| 56 | Methyl benzoate | 18.37 | 1096 | MS,RI | Floral, fruity | nd | 9.38 | 0.12 | 0.0015 |
| 57 | Linalool | 18.69 | 1103 | MS,RI,S | Floral, sweet | 52.10 | 1.39 | 26.29 | 0.0024 |
| 58 | Phenylethyl Alcohol | 19.79 | 1126 | MS,RI,S | Floral, rose-like | 0.67 | 3.08 | 1.02 | 0.021 |
| 59 | 2-Fenchanol / Fenchyl alcohol | 19.97 | 1129 | MS,RI | Spicy, citrus, camphor-like, lemon-like | 0.01 | nd | nd | 0.14 |
| 60 | (4E,6Z)-allo-Ocimene | 20.07 | 1131 | MS,RI | Sweet, floral, nutty, herbal, peppery | 0.04 | 0.08 | 0.18 | 0.01 |
| 61 | Cosmene | 20.13 | 1133 | MS,RI | Herb, citrus | 0.03 | 0.26 | 0.33 | n.f. |
| 62 | Benzyl nitrile | 20.61 | 1143 | MS,RI | Almond-like | 0.03 | 0.38 | 0.07 | 1200 |
| 63 | 2-Norbornanol | 21.38 | 1159 | MS,RI | Camphor-like | 0.03 | nd | nd | n.f. |
| 64 | Benzoic acid | 21.51 | 1161 | MS,RI | Benzene-like, methanal-like | nd | nd | 0.09 | 1000 |
| 65 | Ethyl benzoate | 21.98 | 1171 | MS,RI | Holly-like, green, sweet | nd | 0.69 | 0.35 | 0.0006 |
| 66 | Linalool oxide (pyranoid) | 22.08 | 1173 | MS,RI,S | Floral, honey-like | 0.26 | nd | 0.11 | 3600 |
| 67 | Isoborneol | 22.16 | 1175 | MS,RI | Camphor-like | 0.17 | nd | nd | 16 |
| 68 | Linalool oxide (pyranoid) | 22.31 | 1178 | MS,RI,S | Floral, honey-like | 0.22 | nd | nd | 5400 |
| 69 | Naphthalene | 22.52 | 1182 | MS,RI | Tarry (strong) | nd | 0.08 | nd | 0.45 |
| 70 | 4-Terpineol | 22.54 | 1183 | MS,RI | Pepper-like, earthy (faint), stale wood-like | 0.06 | nd | nd | 0.86 |
| 71 | 3-Methylacetophenone | 22.71 | 1186 | MS,RI | Sweet, fruity, nutty, vanilla-like | 0.01 | nd | nd | 1990 |
| 72 | Methyl salicylate | 22.87 | 1189 | MS,RI,S | Peppermint, minty, fresh, sweet | nd | nd | 0.00 | 0.016 |
| 73 | Ethyl (Z)-4-octenoate | 22.90 | 1190 | MS,RI | Fruity, apple-like, sweet | 0.01 | nd | nd | 50 |
| 74 | 2,6-Dimethyl-3,7-octadiene-2,6-diol | 23.02 | 1193 | MS,RI | Fresh, floral, citrus, sweet | 0.07 | nd | nd | n.f. |
| 75 | *α*-Terpineol | 23.24 | 1197 | MS,RI,S | Pleasant, floral | 0.22 | nd | nd | 0.86 |
| 76 | Estragole | 23.31 | 1199 | MS,RI | Anise-like | 0.24 | 0.15 | 0.80 | 0.00013 |
| 77 | Decanal | 23.76 | 1208 | MS,RI | Sweet, citrus, waxy, floral | 0.01 | nd | nd | 0.0026 |
| 78 | Pulegone | 24.23 | 1218 | MS,RI | Minty, herb, oregano-like | 0.01 | nd | nd | 0.00187 |
| 79 | Nerol | 24.57 | 1226 | MS,RI,S | Floral, fresh, citrus | 0.03 | nd | nd | 0.049 |
| 80 | Citronellol | 24.83 | 1231 | MS,RI | Fresh, rose-like | nd | 0.43 | nd | 0.0465 |
| 81 | Hexyl 2-methylbutanoate | 25.11 | 1237 | MS,RI | Green, waxy, fruity | 0.00 | nd | nd | 22 |
| 82 | Citral | 25.17 | 1239 | MS,RI,S | Lemon-like, citrus | 0.00 | nd | nd | 0.00015 |
| 83 | Geraniol | 25.77 | 1252 | MS,RI,S | Rose-like, sweet, honey-like | 0.06 | nd | nd | 0.6 |
| 84 | Phenethyl acetate | 25.89 | 1254 | MS,RI | Rose-like, honey-like, floral | 0.02 | 0.27 | nd | 249.59 |
| 85 | 2-Phenyl-2-butenal | 26.50 | 1267 | MS,RI | Musty, floral, green, woody | 0.01 | nd | nd | n.f. |
| 86 | Nonanoic acid | 26.71 | 1272 | MS,RI | Fatty (faint), coconut-like | 0.01 | nd | nd | 0.12 |
| 87 | (*Z*)-3-Nonenyl acetate | 26.92 | 1276 | MS,RI | Green, herbaceous, pear-like, tropical | 0.08 | nd | nd | 60 |
| 88 | Bornyl acetate | 27.24 | 1283 | MS,RI | Cooling, pine-like, camphor-like | 0.02 | nd | nd | 0.44 |
| 89 | Anethole | 27.34 | 1285 | MS,RI | Anise-like | 0.01 | 0.24 | 0.75 | 0.057 |
| 90 | Safrole | 27.42 | 1287 | MS,RI | Camphor wood-like | nd | nd | 0.44 | 30 |
| 91 | *cis*-Isosafrole | 27.47 | 1288 | MS,RI | Camphor wood-like | 0.08 | nd | nd | 30 |
| 92 | Indole | 27.56 | 1290 | MS,RI,S | Floral, animal-like | 0.01 | 44.23 | 1.84 | 0.0081 |
| 93 | 2-Undecanone | 27.69 | 1293 | MS,RI | Citrus, rue oil-like | 0.02 | nd | nd | 3 |
| 94 | 2-Methylbenzyl acetate | 27.88 | 1297 | MS,RI | Floral, citrus | 0.01 | nd | nd | 135 |
| 95 | 2-Methoxy-4-vinylphenol | 28.36 | 1308 | MS,RI | Spicy, clove-like, fermentation-like, roasted peanutty | 0.01 | nd | nd | 0.0028 |
| 96 | (*E,E*)-2,4-Decadienal | 28.83 | 1319 | MS,RI,S | Oily, cucumber, fatty, fried | 0.01 | nd | 0.05 | 0.0023 |
| 97 | *δ*-Eiemene | 29.46 | 1333 | MS,RI | Spicy, anise-like | 0.01 | 0.49 | nd | n.f. |
| 98 | Methyl anthranilate | 29.69 | 1338 | MS,RI | Fruity, concord grape | 0.04 | 0.31 | 0.10 | 0.000006 |
| 99 | *α*-Cubebene | 29.98 | 1345 | MS,RI | Spicy, citrus | 0.28 | 0.20 | 0.22 | n.f. |
| 100 | Phenethyl propanoate | 30.16 | 1349 | MS,RI | Sweet, rose-like, fruity, honey-like, strawberry-like | nd | 0.54 | nd | 18 |
| 101 | Eugenol | 30.19 | 1350 | MS,RI,S | Sweet, spicy, clove-like, woody | nd | 0.43 | nd | 0.00061 |
| 102 | Ylangene | 30.26 | 1351 | MS,RI | Floral, citrus | 0.04 | nd | nd | n.f. |
| 103 | *α*-Longipinene | 30.49 | 1357 | MS,RI | Citrus, pine-like | 0.01 | nd | nd | 0.92 |
| 104 | *α*-ylangene | 30.91 | 1366 | MS,RI | Floral, citrus | 0.04 | nd | nd | n.f. |
| 105 | 2,6-Dimethyl-2,7-octadiene-1,6-diol / 8-Hydroxylinalool | 31.03 | 1369 | MS,RI | Floral, sweet | 0.01 | nd | nd | 0.0024 |
| 106 | Copaene | 31.22 | 1373 | MS,RI | Spicy | 0.91 | 0.18 | 0.22 | 0.1 |
| 107 | (*E*)-Isosafrole | 31.35 | 1376 | MS,RI | Camphor wood-like | nd | nd | 0.10 | 30 |
| 108 | *β*-Elemene | 31.51 | 1380 | MS,RI | Anise-like, spicy | 0.10 | 0.91 | 2.74 | n.f. |
| 109 | 3-Methyl-1H-indole | 31.60 | 1382 | MS,RI | Animal-like | nd | 0.28 | nd | 0.00003 |
| 110 | *β*-Cubebene | 31.76 | 1386 | MS,RI | Spicy, citrus | 0.43 | nd | nd | n.f. |
| 111 | 1-Ethenyl-1-methyl-2,4-bis(1-methylethenyl)cyclohexane | 31.90 | 1389 | MS,RI | —— | 2.35 | nd | nd | n.f. |
| 112 | (*Z*)-Jasmone | 32.00 | 1391 | MS,RI | Jasmine-like, herbal, floral, woody | 0.03 | nd | nd | 0.002 |
| 113 | Vanillin | 32.08 | 1393 | MS,RI | Vanilla-like, milky | nd | nd | 0.10 | 0.0012 |
| 114 | Methyleugenol | 32.46 | 1401 | MS,RI | Clove-like, anise-like, carnation-like | 1.81 | 0.44 | 5.35 | 8500 |
| 115 | *α*-Cedrene | 32.90 | 1412 | MS,RI | Woody | 0.08 | nd | nd | n.f. |
| 116 | Caryophyllene | 33.16 | 1418 | MS,RI | Woody, green, spicy, terpenic | 4.17 | 0.43 | 1.57 | 13 |
| 117 | *β*-Gurjunene | 33.48 | 1426 | MS,RI | Smoky (soft) | 0.01 | 0.45 | nd | n.f. |
| 118 | *β*-copaene | 33.50 | 1427 | MS,RI | Spicy | 0.06 | 0.07 | 0.05 | 0.1 |
| 119 | *α*-Bergamotene | 33.69 | 1431 | MS,RI | Lemon-like, citrus | 0.41 | nd | 0.14 | n.f. |
| 120 | *β*-Phenylethyl butyrate | 33.98 | 1438 | MS,RI | Sweet, floral | nd | 0.21 | nd | 87 |
| 121 | Aristolene | 34.00 | 1439 | MS,RI | Woody | 0.05 | nd | nd | n.f. |
| 122 | Alloaromadendrene | 34.09 | 1441 | MS,RI | Floral | 0.01 | nd | nd | n.f. |
| 123 | *epi*-Bicyclosesquiphellandrene | 34.14 | 1442 | MS,RI | Spicy, green, celery-like | nd | 0.14 | nd | n.f. |
| 124 | *α*-Muurolene | 34.17 | 1443 | MS,RI | Ylang-like, fragrance | 0.02 | nd | nd | 0.002 |
| 125 | *trans*-Isoeugenol | 34.27 | 1445 | MS,RI,S | Clove-like, woody, spicy, sweet | nd | 0.30 | nd | 0.006 |
| 126 | *α*-Phenylbutyric acid | 34.28 | 1445 | MS,RI | Aromatic | nd | nd | 0.05 | n.f. |
| 127 | *cis*-Muurola-3,5-diene | 34.30 | 1446 | MS,RI | Floral, fragrance | 0.12 | nd | nd | n.f. |
| 128 | (*E*)-*β*-Famesene | 34.55 | 1452 | MS,RI | Green, floral, balsam-like | nd | 0.60 | nd | 87 |
| 129 | Humulene | 34.59 | 1453 | MS,RI | Woody | 1.30 | nd | 0.71 | 160 |
| 130 | *β*-Santalene | 34.74 | 1456 | MS,RI | Sandalwood-like, woody | 0.09 | nd | nd | 30 |
| 131 | *γ*-Muurolene | 34.83 | 1458 | MS,RI | Floral, fragrance, Ylang-like | 0.03 | 0.10 | nd | n.f. |
| 132 | *γ*-Gurjunene | 35.06 | 1464 | MS,RI | Smoky | 0.06 | nd | 0.09 | n.f. |
| 133 | (*E*)-Ethyl cinnamate | 35.12 | 1465 | MS,RI | Fruity, fragrance, sweet | nd | nd | 0.10 | 165 |
| 134 | 1-Methyl-4-methylene-2-(2-methyl-1-propenyl)-1-vinylcycloheptane | 35.18 | 1467 | MS,RI | —— | 0.04 | nd | nd | n.f. |
| 135 | 2-Isopropenyl-4a,8-dimethyl-1,2,3,4,4a,5,6,8a-octahydronaphthalene | 35.31 | 1470 | MS,RI | —— | 0.16 | nd | 0.03 | n.f. |
| 136 | *α*-Elemene | 35.40 | 1472 | MS,RI | Anise-like, spicy | 0.15 | 0.65 | 0.19 | n.f. |
| 137 | Germacrene D | 35.64 | 1478 | MS,RI | Woody, spicy | 0.96 | 0.34 | 0.59 | n.f. |
| 138 | *α*-Curcumene | 35.66 | 1478 | MS,RI | Spicy, ginger-like | nd | 0.31 | 0.33 | n.f. |
| 139 | *cis*-*β*-Farnesene | 35.79 | 1481 | MS,RI,S | Floral, citrus | 0.21 | nd | 0.10 | 87 |
| 140 | Phenylethyl 2-methylbutanoate | 35.86 | 1483 | MS,RI | Rose-like, fruity | nd | 4.44 | 1.71 | 12 |
| 141 | *β*-Selinene | 35.96 | 1485 | MS,RI | Celery-like | 0.85 | nd | nd | 1 |
| 142 | 2-Isopropyl-5-methyl-9-methylenebicyclo[4.4.0]dec-1-ene | 36.06 | 1488 | MS,RI | —— | 0.11 | nd | nd | n.f. |
| 143 | *α*-Zingiberene | 36.16 | 1490 | MS,RI | Ginger-like | 0.11 | nd | nd | n.f. |
| 144 | *α*-Selinene | 36.25 | 1493 | MS,RI | Celery-like | 0.70 | 0.53 | 1.02 | 1 |
| 145 | Methylisoeugenol | 36.34 | 1495 | MS,RI | Eugenol-like, sweet, spicy, woody | 0.41 | 1.39 | 11.76 | 1600 |
| 146 | *α*-Guaiene | 36.45 | 1497 | MS,RI | Earthy, spicy | nd | 0.22 | nd | n.f. |
| 147 | *α*-Bulnesene | 36.51 | 1499 | MS,RI | Herbal, spicy, green | 0.18 | nd | 0.27 | n.f. |
| 148 | *α*-Farnesene | 36.67 | 1503 | MS,RI,S | Woody, green, floral, herbal | nd | 0.12 | 0.51 | 87 |
| 149 | Eremophilene | 36.74 | 1505 | MS,RI | —— | 1.33 | nd | nd | n.f. |
| 150 | *β*-Bisabolene | 36.80 | 1506 | MS,RI | Woody, citrus, floral, fruity, green, balsam | 0.29 | 0.98 | 1.07 | n.f. |
| 151 | *γ*-Cadinene | 36.93 | 1509 | MS,RI | Fruity, sweet, sour, woody, syrup-like, strawberry-like | 0.04 | 0.74 | 0.14 | n.f. |
| 152 | *β*-Cadinene | 37.05 | 1512 | MS,RI | Minty, camphoraceous, herbaceous, woody, phenolic, warm | 0.04 | 0.57 | nd | n.f. |
| 153 | *δ*-Cadinene | 37.19 | 1516 | MS,RI | Herbal, woody | 1.06 | nd | 1.52 | 0.222 |
| 154 | *trans*-calamenene | 37.25 | 1517 | MS,RI | Herbal, spicy | nd | 0.71 | nd | 2 |
| 155 | *cis*-Calamenene | 37.28 | 1518 | MS,RI | Citrus (faint), green | 0.05 | 1.00 | 0.18 | 2 |
| 156 | *β*-Sesquiphellandrene | 37.41 | 1521 | MS,RI | Celery-like | 0.03 | 0.18 | 0.09 | 500 |
| 157 | 1,2,3,4,4a,7-Hexahydro-1,6-dimethyl-4-(1-methylethyl)naphthalene | 37.71 | 1529 | MS,RI | —— | 0.05 | 0.14 | 0.14 | 2 |
| 158 | *α*-Cadinene | 37.84 | 1532 | MS,RI | Minty, camphoraceous, herbaceous, woody, phenolic, warm | nd | 0.35 | nd | n.f. |
| 159 | *γ*-Selinene | 37.88 | 1533 | MS,RI | Celery-like, green, spicy | 0.02 | nd | nd | 1 |
| 160 | *α*-Calacorene | 38.04 | 1537 | MS,RI | Woody | 0.04 | 0.97 | 0.55 | n.f. |
| 161 | 2-Methoxy-1-(2-methyl-1-propenyl)indane | 38.13 | 1540 | MS,RI | —— | nd | 0.08 | 0.05 | ＞510 |
| 162 | Elemicine | 38.38 | 1546 | MS,RI | Spicy | 0.04 | 0.08 | 0.11 | 10000 |
| 163 | Germacrene B | 38.70 | 1554 | MS,RI | Tobacco-like, tea-like, licorice-like | 0.02 | nd | nd | n.f. |
| 164 | Cadala-1(10),3,8-triene | 38.84 | 1558 | MS,RI | Fresh, woody | nd | 0.13 | nd | n.f. |
| 165 | Nerolidol | 38.93 | 1560 | MS,RI,S | Floral, green, citrus, woody, waxy | 0.15 | nd | 0.55 | 10000 |
| 166 | 1,6,7-Trimethylnaphthalene | 39.30 | 1569 | MS,RI | Aromatic | nd | 0.21 | nd | 2.5 |
| 167 | Espatulenol | 39.43 | 1573 | MS,RI | Cinnamon-like, spicy | 0.01 | nd | 0.05 | n.f. |
| 168 | Hexyl benzoate | 39.58 | 1577 | MS,RI | Fresh, balsam, sappy | nd | 0.10 | nd | 0.0006 |
| 169 | Caryophyllene oxide | 39.62 | 1577 | MS,RI | Woody | 0.25 | nd | 0.35 | 410 |
| 170 | 2-Phenylethyl tiglate | 39.74 | 1581 | MS,RI | Berry, caramel-like, floral, tropical, fruity, sweet | nd | 0.40 | nd | 65 |
| 171 | Isoaromadendrene epoxide | 40.03 | 1588 | MS,RI | Citrus, fruity | 0.01 | nd | nd | n.f. |
| 172 | 1-Methyl-8-(1-methylethyl)tricyclo[4.4.0.0(2,7)]dec-3-ene-3-methanol | 40.35 | 1596 | MS,RI | —— | 0.01 | nd | nd | n.f. |
| 173 | Hexadecane | 40.50 | 1600 | MS,RI,S | Alkane-like | nd | 0.08 | nd | 0.5 |
| 174 | Humulene oxide II | 40.69 | 1605 | MS,RI | Spicy | 0.07 | nd | nd | 10 |
| 175 | *epi*-Cadinol | 41.96 | 1639 | MS,RI | Pine-like, spicy | 0.04 | nd | nd | 200000 |
| 176 | Methyl jasmonate | 41.99 | 1640 | MS,RI,S | Jasmine-like, floral | nd | 0.40 | nd | 5700 |
| 177 | *epi*-*α*-Muurolol | 42.02 | 1640 | MS,RI | Floral, sweet | 0.09 | nd | 0.49 | 200000 |
| 178 | *α*-Cadinol | 42.44 | 1652 | MS,RI | Woody, herbal | 0.02 | nd | nd | 200000 |
| 179 | (*Z*)-4-Hexadecen-6-yne | 42.66 | 1658 | MS,RI | —— | nd | nd | 0.28 | n.f. |
| 180 | Decahydro-5-methylene-8-vinyl-2-naphthalenemethanol | 42.97 | 1666 | MS,RI | —— | nd | nd | 0.41 | n.f. |
| 181 | Cadalene | 43.04 | 1668 | MS,RI | Coal tar-like | nd | 0.09 | 0.37 | 10 |
| 182 | *α*-Sinensal | 43.51 | 1680 | MS,RI | Citrus, tropical | nd | nd | 0.05 | 220 |
| 183 | Aromadendrene oxide-(2) | 43.66 | 1684 | MS,RI | Citrus | nd | nd | 0.07 | n.f. |
| 184 | (*Z,E*)-Farnesol | 44.00 | 1694 | MS,RI | Limette-like | nd | 0.17 | nd | 1000 |
| 185 | Heptadecane | 44.23 | 1700 | MS,RI,S | Alkane-like | nd | 0.16 | nd | 10000000 |
| 186 | Cedrenol | 44.69 | 1712 | MS,RI | Woody, sweet cream | 0.01 | nd | nd | n.f. |
| 187 | Farnesol | 44.81 | 1716 | MS,RI | Floral, fresh, sweet | nd | 0.08 | nd | 1000 |
| 188 | Methyl tetradecanoate | 45.10 | 1724 | MS,RI | Fatty, waxy | nd | 0.12 | nd | 0.5 |
| 189 | Nootkatone | 46.53 | 1764 | MS,RI | Citrus, grapefruit-like | nd | nd | 0.54 | 280 |
| 190 | Guaiazulene | 46.64 | 1767 | MS,RI | —— | nd | 0.08 | nd | n.f. |
| 191 | Octadecane | 47.80 | 1800 | MS,RI,S | Alkane-like | nd | 0.23 | nd | 0.02 |
| 192 | 9-Nonadecene | 50.42 | 1877 | MS,RI | Alkane-like | nd | 0.53 | nd | n.f. |
| 193 | Nonadecane | 51.21 | 1900 | MS,RI,S | Alkane-like | 0.01 | 3.09 | nd | 10000000 |
| 194 | Methyl hexadecanoate | 52.02 | 1924 | MS,RI | Oily, waxy, fatty | 0.00 | 4.60 | nd | ＞2000 |
| 195 | *n*-Hexadecanoic acid | 53.21 | 1958 | MS,RI | Waxy, creamy, candle-like | 0.00 | nd | 0.37 | ＞10000 |
| 196 | Ethyl hexadecanoate | 54.46 | 1994 | MS,RI | Oily, waxy, fatty | 0.03 | 0.05 | 4.71 | 2000 |
| 197 | *E*-15-Heptadecenal | 56.66 | 2081 | MS,RI | Grassy, green, pungent smelly | nd | 0.12 | nd | n.f. |
| 198 | Methyl linoleate | 56.93 | 2092 | MS,RI | Oily, fatty, woody | 0.00 | 2.98 | 0.72 | 450 |
| 199 | Methyl linolenate | 57.03 | 2096 | MS,RI | Fatty, waxy | nd | 1.54 | nd | 450 |
| 200 | Heneicosane | 57.14 | 2100 | MS,RI | Alkane-like | 0.01 | 0.93 | nd | 10000000 |
| 201 | Methyl (*Z,Z*)-9,15-octadecadienoate | 57.90 | 2144 | MS,RI | Fatty, waxy | nd | 0.19 | nd | 450 |
| 202 | Ethyl linoleate | 58.15 | 2159 | MS,RI | Oily, fatty, woody | 0.11 | nd | 3.25 | 450 |
| 203 | Ethyl linolenate | 58.25 | 2164 | MS,RI | Fatty, waxy | 0.07 | nd | 2.66 | 450 |
| 204 | Butyl hexadecanoate | 58.63 | 2186 | MS,RI | Waxy | 0.03 | nd | nd | ＞2000 |
| 205 | Ethyl octadecanoate | 58.76 | 2193 | MS,RI | Waxy | 0.00 | nd | nd | ＞500 |
| 206 | Tricosane | 60.84 | 2301 | MS,RI,S | Alkane-like | 0.01 | 0.20 | 0.24 | 10000000 |
| 207 | Butyl octadecanoate | 62.37 | 2389 | MS,RI | Waxy | 0.06 | nd | nd | ＞500 |
| ^ζ^ Identification method. MS, identification based on the NIST 2017 mass spectral database; RI, retention index; S, the compounds were identified using authentic standard compounds. | | | | | | | | | |
| ^ψ^ Odor description found in the literature (Flavornet; The LRI and Odour Database). | | | | | | | | | |
| '——', no odor description information was found in the literature. | | | | | | | | | |
| nd, not detectable. | | | | | | | | | |
| 'n.f.', data was not found in the literature. | | | | | | | | | |

**Table S2** The rOAV values of identified volatiles in white champaca from fresh and dried flowers

| Volatile compounds | rOAV (%) | | |
| --- | --- | --- | --- |
|  | DF-HS | FF-SPME | DF-SPME |
| Butanal | 0.01 | nd | nd |
| 2,3-Butanedione | 0.05 | nd | nd |
| Acetic acid | 0.14 | nd | nd |
| Ethyl Acetate | nd | nd | 0.00 |
| 2-Methylfuran | 0.00 | nd | nd |
| (*E*)-2-Butenal | nd | nd | 0.00 |
| 3-Methylbutanal | 4.43 | nd | nd |
| 2-Methylbutanal | 0.05 | nd | nd |
| 2-Ethylfuran | 0.00 | nd | nd |
| Ethyl propanoate | 0.00 | nd | nd |
| 1-Methoxypentane | nd | nd | nd |
| Ethyl 2-methylpropanoate | 88.98 | nd | nd |
| Toluene | 0.00 | nd | nd |
| Methyl 2-methylbutanoate | 0.00 | nd | nd |
| 3-Methyl-2-butenal | 0.00 | nd | nd |
| Hexanal | 0.01 | nd | 0.00 |
| Methylpent-4-enylamine | nd | nd | nd |
| Methyl 2,3-dimethylbutanoate | nd | nd | nd |
| Furfural | 0.00 | nd | nd |
| Methyl valerate | 0.00 | nd | nd |
| Ethyl 2-methylbutanoate | 0.00 | nd | 0.00 |
| 2-Hexenal | nd | nd | 0.00 |
| 2-Methylbutanoic acid | 0.24 | nd | 0.78 |
| Methyl tiglate | nd | 0.00 | nd |
| 2-Heptanone | 0.83 | nd | nd |
| (*E,E*)-2,4-Hexadienal | nd | nd | 0.65 |
| Pentanoic acid | 2.84 | nd | nd |
| 2-Methylhexanoic acid | 0.00 | nd | nd |
| Butyrolactone | 0.00 | nd | nd |
| *α*-Pinene | 0.04 | nd | nd |
| Ethyl 2-methyl-2-butenoate | 0.00 | nd | nd |
| Camphene | 0.00 | nd | nd |
| Benzaldehyde | 0.01 | 0.01 | 0.03 |
| *β*-Thujene | nd | nd | nd |
| *β*-Pinene | 0.02 | nd | nd |
| 6-Methyl-5-hepten-2-one | 0.01 | nd | nd |
| *β*-Myrcene | 0.01 | nd | 0.01 |
| Hexanoic acid | 0.08 | nd | nd |
| Ethyl hexanoate | 0.00 | nd | 0.00 |
| *α*-Phellandrene | 0.00 | nd | nd |
| (*E,E*)-2,4-Heptadienal | nd | nd | 0.02 |
| 3-Pyridinecarbonitrile | nd | nd | nd |
| *α*-Terpinene | 0.00 | nd | nd |
| *o*-Cymene | 0.16 | nd | nd |
| Limonene | 0.01 | nd | 0.00 |
| Eucalyptol | 0.05 | nd | 0.01 |
| *trans*-*β*-Ocimene | 0.78 | nd | 0.10 |
| Benzeneacetaldehyde | 0.00 | nd | 0.00 |
| *α*-Ocimene | 1.07 | 0.04 | 0.33 |
| *γ*-Terpinene | 0.00 | nd | nd |
| Linalool oxide 2 (trans, furanoid) | 0.00 | nd | nd |
| 1-Octanol | nd | 0.00 | nd |
| 1-Phenyl-1-butene | nd | nd | 0.00 |
| *α*-Terpinolen | 0.00 | nd | nd |
| Linalool oxide 1 (cis, furanoid) | 0.00 | nd | 0.00 |
| Methyl benzoate | nd | 12.13 | 0.46 |
| Linalool | 100.00 | 1.12 | 65.58 |
| Phenylethyl Alcohol | 0.15 | 0.28 | 0.29 |
| 2-Fenchanol / Fenchyl alcohol | 0.00 | nd | nd |
| (*E,Z*)-2,6-Dimethyl-2,4,6-octatriene / (4E,6Z)-allo-Ocimene | 0.02 | 0.02 | 0.11 |
| Cosmene | nd | nd | nd |
| Benzyl nitrile / Phenylacetonitrile | 0.00 | 0.00 | 0.00 |
| 2-Norbornanol | nd | nd | nd |
| Benzoic acid | nd | nd | 0.00 |
| Ethyl benzoate | nd | 2.22 | 3.47 |
| Linalool oxide (pyranoid) | 0.00 | nd | 0.00 |
| Isoborneol | 0.00 | nd | nd |
| Linalool oxide (pyranoid) | 0.00 | nd | nd |
| Naphthalene | nd | 0.00 | nd |
| 4-Terpineol | 0.00 | nd | nd |
| 3-Methylacetophenone | 0.00 | nd | nd |
| Methyl salicylate | nd | nd | 0.00 |
| Ethyl (Z)-4-octenoate | 0.00 | nd | nd |
| 2,6-Dimethyl-3,7-octadiene-2,6-diol | nd | nd | nd |
| *α*-Terpineol | 0.00 | nd | nd |
| Estragole | 8.43 | 2.22 | 36.77 |
| Decanal | 0.01 | nd | nd |
| Pulegone | 0.03 | nd | nd |
| Nerol | 0.00 | nd | nd |
| Citronellol | nd | 0.02 | nd |
| Hexyl 2-methylbutanoate | 0.00 | nd | nd |
| Citral | 0.08 | nd | nd |
| Geraniol | 0.00 | nd | nd |
| Phenethyl acetate | 0.00 | 0.00 | nd |
| 2-Phenyl-2-butenal | nd | nd | nd |
| Nonanoic acid | 0.00 | nd | nd |
| (*Z*)-3-Nonenyl acetate | 0.00 | nd | nd |
| Bornyl acetate | 0.00 | nd | nd |
| Anethole | 0.00 | 0.01 | 0.08 |
| Safrole | nd | nd | 0.00 |
| *cis*-Isosafrole | 0.00 | nd | nd |
| Indole | 0.01 | 10.60 | 1.36 |
| 2-Undecanone | 0.00 | nd | nd |
| 2-Methylbenzyl acetate | 0.00 | nd | nd |
| 2-Methoxy-4-vinylphenol | 0.02 | nd | nd |
| (*E,E*)-2,4-Decadienal | 0.01 | nd | 0.12 |
| *δ*-Eiemene | nd | nd | nd |
| Methyl anthranilate | 29.48 | 100.00 | 100.00 |
| *α*-Cubebene | nd | nd | nd |
| Phenethyl propanoate | nd | 0.00 | nd |
| Eugenol | nd | 1.38 | nd |
| Ylangene | nd | nd | nd |
| *α*-Longipinene | 0.00 | nd | nd |
| *α*-ylangene | nd | nd | nd |
| 2,6-Dimethyl-2,7-octadiene-1,6-diol / 8-Hydroxylinalool | 0.02 | nd | nd |
| Copaene | 0.04 | 0.00 | 0.01 |
| (*E*)-Isosafrole | nd | nd | 0.00 |
| *β*-Elemene | nd | nd | nd |
| 3-Methyl-1H-indole | nd | 18.29 | nd |
| *β*-Cubebene | nd | nd | nd |
| 1-Ethenyl-1-methyl-2,4-bis(1-methylethenyl)cyclohexane | nd | nd | nd |
| (*Z*)-Jasmone | 0.06 | nd | nd |
| Vanillin | nd | nd | 0.49 |
| Methyleugenol | 0.00 | 0.00 | 0.00 |
| *α*-Cedrene | nd | nd | nd |
| Caryophyllene | 0.00 | 0.00 | 0.00 |
| *β*-Gurjunene | nd | nd | nd |
| *β*-copaene | 0.00 | 0.00 | 0.00 |
| *α*-Bergamotene | nd | nd | nd |
| *β*-Phenylethyl butyrate | nd | 0.00 | nd |
| Aristolene | nd | nd | nd |
| Alloaromadendrene | nd | nd | nd |
| *epi*-Bicyclosesquiphellandrene | nd | nd | nd |
| *α*-Muurolene | 0.04 | nd | nd |
| *trans*-Isoeugenol | nd | 0.10 | nd |
| *α*-Phenylbutyric acid | nd | nd | nd |
| *cis*-Muurola-3,5-diene | nd | nd | nd |
| (*E*)-*β*-Famesene | nd | 0.00 | nd |
| Humulene | 0.00 | nd | 0.00 |
| *β*-Santalene | 0.00 | nd | nd |
| *γ*-Muurolene | nd | nd | nd |
| *γ*-Gurjunene | nd | nd | nd |
| (*E*)-Ethyl cinnamate | nd | nd | 0.00 |
| 1-Methyl-4-methylene-2-(2-methyl-1-propenyl)-1-vinylcycloheptane | nd | nd | nd |
| 2-Isopropenyl-4a,8-dimethyl-1,2,3,4,4a,5,6,8a-octahydronaphthalene | nd | nd | nd |
| *α*-Elemene | nd | nd | nd |
| Germacrene D | nd | nd | nd |
| *α*-Curcumene | nd | nd | nd |
| *cis*-*β*-Farnesene | 0.00 | nd | 0.00 |
| Phenylethyl 2-methylbutanoate | nd | 0.00 | 0.00 |
| *β*-Selinene | 0.00 | nd | nd |
| 2-Isopropyl-5-methyl-9-methylenebicyclo[4.4.0]dec-1-ene | nd | nd | nd |
| *α*-Zingiberene | nd | nd | nd |
| *α*-Selinene | 0.00 | 0.00 | 0.01 |
| Methylisoeugenol | 0.00 | 0.00 | 0.00 |
| *α*-Guaiene | nd | nd | nd |
| *α*-Bulnesene | nd | nd | nd |
| *α*-Farnesene | nd | 0.00 | 0.00 |
| Eremophilene | nd | nd | nd |
| *β*-Bisabolene | nd | nd | nd |
| *γ*-Cadinene | nd | nd | nd |
| *β*-Cadinene | nd | nd | nd |
| *δ*-Cadinene | 0.02 | nd | 0.04 |
| *trans*-calamenene | nd | 0.00 | nd |
| *cis*-Calamenene | 0.00 | 0.00 | 0.00 |
| *β*-Sesquiphellandrene | 0.00 | 0.00 | 0.00 |
| 1,2,3,4,4a,7-Hexahydro-1,6-dimethyl-4-(1-methylethyl)naphthalene | 0.00 | 0.00 | 0.00 |
| *α*-Cadinene | nd | nd | nd |
| *γ*-Selinene | 0.00 | nd | nd |
| *α*-Calacorene | nd | nd | nd |
| 2-Methoxy-1-(2-methyl-1-propenyl)indane | nd | 0.00 | 0.00 |
| Elemicine | 0.00 | 0.00 | 0.00 |
| Germacrene B | nd | nd | nd |
| Cadala-1(10),3,8-triene | nd | nd | nd |
| Nerolidol | 0.00 | nd | 0.00 |
| 1,6,7-Trimethylnaphthalene | nd | 0.00 | nd |
| Espatulenol | nd | nd | nd |
| Hexyl benzoate | nd | 0.34 | nd |
| Caryophyllene oxide | 0.00 | nd | 0.00 |
| 2-Phenylethyl tiglate | nd | 0.00 | nd |
| Isoaromadendrene epoxide | nd | nd | nd |
| 1-Methyl-8-(1-methylethyl)tricyclo[4.4.0.0(2,7)]dec-3-ene-3-methanol | nd | nd | nd |
| Hexadecane | nd | 0.00 | nd |
| Humulene oxide II | 0.00 | nd | nd |
| *epi*-Cadinol | 0.00 | nd | nd |
| Methyl jasmonate | nd | 0.00 | nd |
| *epi*-*α*-Muurolol | 0.00 | nd | 0.00 |
| *α*-Cadinol | 0.00 | nd | nd |
| (*Z*)-4-Hexadecen-6-yne | nd | nd | nd |
| Decahydro-5-methylene-8-vinyl-2-naphthalenemethanol | nd | nd | nd |
| Cadalene | nd | 0.00 | 0.00 |
| *α*-Sinensal | nd | nd | 0.00 |
| Aromadendrene oxide-(2) | nd | nd | nd |
| (*Z,E*)-Farnesol | nd | 0.00 | nd |
| Heptadecane | nd | 0.00 | nd |
| Cedrenol | nd | nd | nd |
| Farnesol | nd | 0.00 | nd |
| Methyl tetradecanoate | nd | 0.00 | nd |
| Nootkatone | nd | nd | 0.00 |
| Guaiazulene | nd | nd | nd |
| Octadecane | nd | 0.02 | nd |
| 9-Nonadecene | nd | nd | nd |
| Nonadecane | 0.00 | 0.00 | nd |
| Methyl hexadecanoate | 0.00 | 0.00 | nd |
| *n*-Hexadecanoic acid | 0.00 | nd | 0.00 |
| Ethyl hexadecanoate | 0.00 | 0.00 | 0.00 |
| *E*-15-Heptadecenal | nd | nd | nd |
| Methyl linoleate | 0.00 | 0.00 | 0.00 |
| Methyl linolenate | nd | 0.00 | nd |
| Heneicosane | 0.00 | 0.00 | nd |
| Methyl (*Z,Z*)-9,15-octadecadienoate | nd | 0.00 | nd |
| Ethyl linoleate | 0.00 | nd | 0.00 |
| Ethyl linolenate | 0.00 | nd | 0.00 |
| Butyl hexadecanoate | 0.00 | nd | nd |
| Ethyl octadecanoate | 0.00 | nd | nd |
| Tricosane | 0.00 | 0.00 | 0.00 |
| Butyl octadecanoate | 0.00 | nd | nd |

nd, not detectable.
